# Genetic and Environmental Control of Neurodevelopmental Robustness in *Drosophila*

**DOI:** 10.1371/journal.pone.0155957

**Published:** 2016-05-25

**Authors:** David J. Mellert, W. Ryan Williamson, Troy R. Shirangi, Gwyneth M. Card, James W. Truman

**Affiliations:** Janelia Research Campus, Howard Hughes Medical Institute, Ashburn, Virginia, United States of America; University of Bern, SWITZERLAND

## Abstract

Interindividual differences in neuronal wiring may contribute to behavioral individuality and affect susceptibility to neurological disorders. To investigate the causes and potential consequences of wiring variation in *Drosophila melanogaster*, we focused on a hemilineage of ventral nerve cord interneurons that exhibits morphological variability. We find that late-born subclasses of the 12A hemilineage are highly sensitive to genetic and environmental variation. Neurons in the second thoracic segment are particularly variable with regard to two developmental decisions, whereas its segmental homologs are more robust. This variability “hotspot” depends on Ultrabithorax expression in the 12A neurons, indicating variability is cell-intrinsic and under genetic control. 12A development is more variable and sensitive to temperature in long-established laboratory strains than in strains recently derived from the wild. Strains with a high frequency of one of the 12A variants also showed a high frequency of animals with delayed spontaneous flight initiation, whereas other wing-related behaviors did not show such a correlation and were thus not overtly affected by 12A variation. These results show that neurodevelopmental robustness is variable and under genetic control in Drosophila and suggest that the fly may serve as a model for identifying conserved gene pathways that stabilize wiring in stressful developmental environments. Moreover, some neuronal lineages are variation hotspots and thus may be more amenable to evolutionary change.

## Introduction

Phenotypic variation is an ubiquitous attribute of biological populations, and identifying its sources and consequences is essential for establishing evolutionary patterns and principles[1,2]. Studies of variation can also reveal properties of the developmental programs that establish a trait [3,4], provide context for comparisons of homologous traits between species [5], and reveal mechanisms that confer robustness [6].

Nervous system development is one area in which studies of variation and robustness will be especially valuable. Understanding how neural networks vary provides realistic constraints for functional models and identifies sources of behavioral individuality, which in turn aids in constructing hypotheses for how neural networks evolve. Alternatively, it is also necessary to understand how systems suppress variation, as developmental robustness prevents deleterious variation in neural network topology. For example, it is proposed that neurological disorders such as schizophrenia and autism owe much of their genetic complexity to gene-gene and gene-environment interactions that are exacerbated in individuals with reduced neurodevelopmental robustness [7–9]. However, little is known about what types of genetic variants may impact neurodevelopmental robustness in natural populations.

We are using *Drosophila melanogaster* to determine to what extent the developmental programs that establish the central nervous system (CNS) are sensitive to genetic and environmental variation and developmental noise, and how the CNS subsequently responds and adapts to these types of variation. Genetic and developmental analyses of *Drosophila* CNS development have found that neuronal fate determination and development are sufficiently precise that single, identifiable neurons can be sequentially produced by a common precursor in a stereotyped birth order [10,11]. Nonetheless, many aspects of development are variable or plastic. Local interneurons in the antennal lobe exhibit variable fine-scale connectivity and physiology [12]. Mushroom body neurons involved in learning and memory show both experience dependent and nutrition dependent plasticity [13,14] and exhibit nondeterministic patterns of connectivity [15]. Visual system neurons can also show plastic responses to experience [16,17]. These types of variation are not necessarily surprising, as they largely occur in sensory and memory systems where plasticity is essential, so structural variability may result from adaptive or homeostatic mechanisms. We are primarily interested in deviations from the developmental programs that produce more “hardwired” circuitry, because these deviations should better reflect variation due to developmental noise (e.g. [18] or genetic variation.

**Fig 1:**
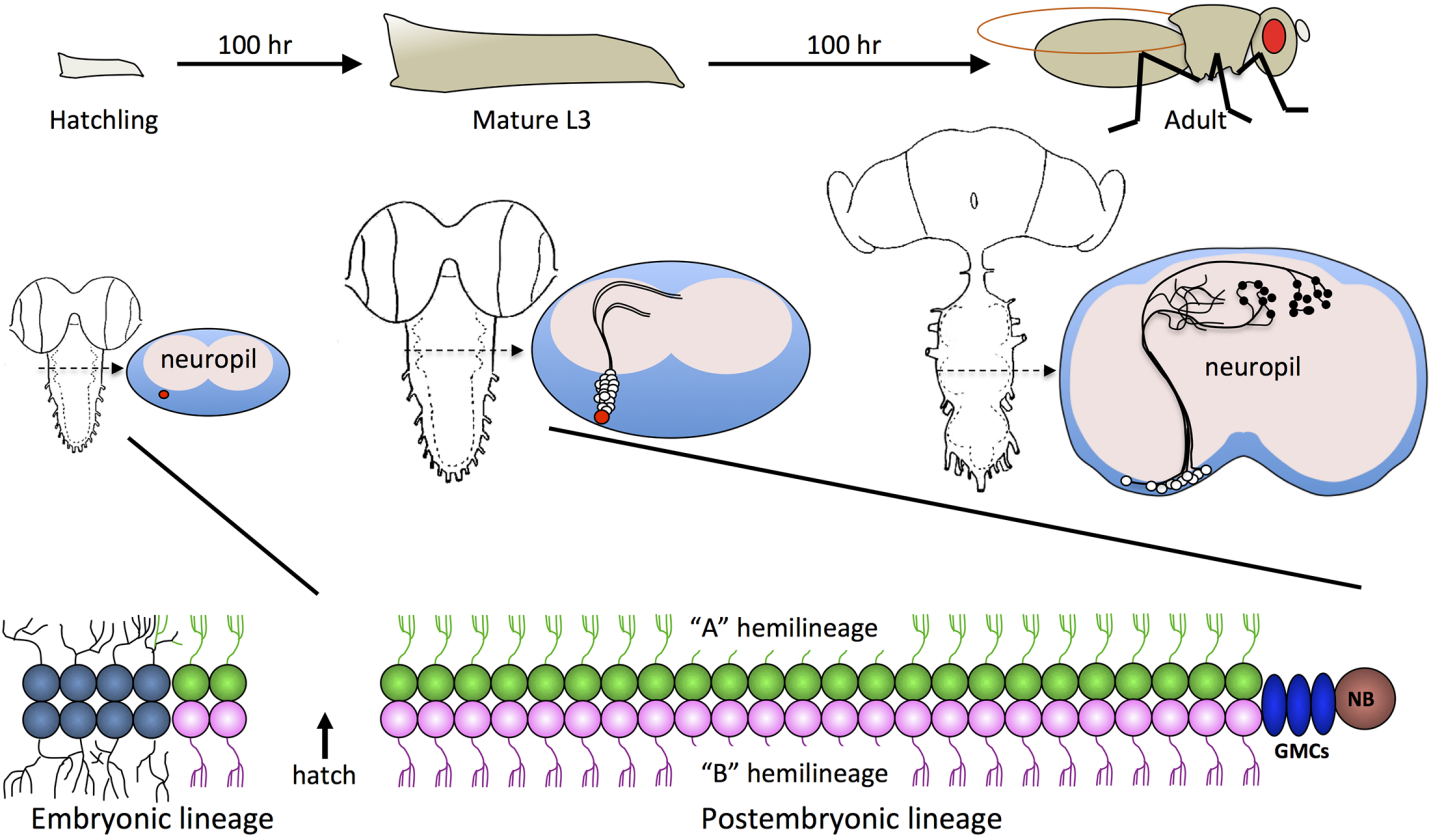
Hemilineage organization of the Drosophila VNC. Top Row: Timeline of Drosophila development. Middle Row: Around hatching (left), the neuroblast (NB; red) switches from producing larval neurons (embryonic lineage) to adult neurons (postembryonic lineage). The NB continues to produce adult neurons throughout larval development, but these neurons produce only primary neurites (center), and then developmentally stall. In the example shown, the neuroblast is producing two hemilineages, hence two primary neurite bundles in which neurites belonging to the same hemilineage cofasciculate. During metamorphosis (right), the adult neurons complete development and form synapses. Bottom Row: Schematic of NBs during the production of larval neurons and adult neurons. Each ganglion mother cell (GMC) produces an ‘A’ cell and a ‘B’ cell, creating two postembryonic hemilineages of adult neurons. For some NBs, one of the two hemilineages undergoes programmed cell death (not shown in schematic).

We have focused on the Drosophila ventral nerve cord (VNC), which contains the circuitry for most motor patterns. In many respects, VNC development is consistent across animals [19,20]. Neurons are produced by neuroblasts, which are arranged in a segmentally repeating array containing 30 per hemisegment [21,22]. Each neuroblast has a unique genetic signature and position within the array [23] and produces a characteristic set of neurons. Neurogenesis results from asymmetric divisions of the neuroblast to produce a series of ganglion mother cells, each of which terminally divides to produce two neurons (Fig 1, bottom row). The pair of neurons produced by each ganglion mother cell differ with respect to Notch signaling [24], so each neuroblast can produce two neuronal populations: Notch-on “A” cells and Notch-off “B” cells. During embryogenesis, neurons are born that contribute to the larval nervous system. A subset of neuroblasts continue dividing throughout larval development to produce neurons for the adult nervous system [20,25], but these cells remain developmentally stalled until the end of the larval growth period (Fig 1, middle row).

The postembryonic neurons inherit their identity both from their parent neuroblast and through Notch signaling, so that each neuroblast produces an “A” and “B” hemilineage, although depending on the neuroblast one hemilineage or the other may be eliminated by programmed cell death (Fig 1) [26]. The immature neurites of neurons within a hemilineage cofasciculate, creating within the late larval VNC a collection of ordered neurite bundles that exhibit hemilineage-specific morphology, suggesting each hemilineage represents a distinct neuronal class. This hypothesis is supported by experiments in which selected neuronal hemilineages were activated in the adult, causing hemilineage-specific behavioral phenotypes [27].

Because most developmental studies have focused on the stereotyped features of the VNC, little is known about the extent of variability and plasticity in its development, connectivity, and function. We focused here on variability within the postembryonic hemilineages, as they constitute the vast majority of neurons in the adult VNC [28]. Our entry point was hemilineage 12A, which was previously noted to be variable [20]. Although developmental studies of the VNC hemilineages have historically required the laborious production of stochastic genetic mosaics, the *R24B02-GAL4* [29,30] driver line specifically targets 12A neurons [30], thereby allowing us to examine numerous examples of the 12A interneurons under varied environmental and genetic conditions. We find a striking sensitivity of hemilineage 12A development to genetic background and environment and that its neurodevelopmental robustness differs between strains. The resulting morphological variation in the 12A neurons is also correlated with delays in flight initiation. This work establishes the Drosophila VNC as a system for identifying genetic variants, and potentially gene pathways, that affect neurodevelopmental robustness. Moreover, the observed patterns of variation provide clues for how neural networks might evolve.

## Materials and Methods

### Preparation and examination of tissues

The projection pattern of the 12A neurite bundles was referenced to tract staining for neuroglian (Ab BP104) [20,31]. Tissues were dissected in PBS and fixed in 4% formaldehyde in PBS (pH 7.0) for 45–60 minutes, then rinsed in PBS-TX (phosphate buffered saline (pH 7.2) with 1% Triton-X100). Antibody staining was performed as described by Truman et al. [20] using the following antibodies:

Primary antibodies: mouse anti-Neuroglian (1:40; Developmental Studies Hybridoma Bank [DSHB] BP104), rat anti-DN-cadherin (1:40; DSHB DN-Ex #8), rabbit anti-GFP (1:1000; Invitrogen), chicken anti-GFP (1:500; Abcam ab13970), rabbit anti-DsRed (1:500; Clontech #632496), and rabbit anti-Ubx (1:500; 7701[32]).

Secondary antibodies: Alexa Fluor^®^ 488-conjugated donkey anti-rabbit (1:500; Invitrogen), Alexa Fluor^®^ 568-conjugated goat anti-mouse (1:500; Invitrogen), Cy5-conjugated Donkey anti-mouse (1:500; Jackson ImmunoResearch Laboratories, Inc.), Alexa Fluor^®^ 488 conjugated goat anti-chicken (1:500; Invitrogen), Texas Red conjugated donkey anti-rat IgG (1:500; Jackson ImmunoResearch Laboratories, Inc.), and Texas Red conjugated Donkey anti-rabbit (1:500; Jackson ImmunoResearch Laboratories, Inc.).

We used a Zeiss LSM 510 confocal microscope for imaging. Z-stacks were analyzed using ImageJ (http://rsb.info.nih.gov/ij/) and Vaa3D [33]. ImageJ and Vaa3D were used for image processing. For publication purposes, transverse optical sections were additionally manipulated in ImageJ to remove out-of-plane signal that would interfere with the max-projected image, but masking was not used to analyze the raw data.

### Ubx Quantification

To quantify Ubx expression, we prepared and imaged anti-Ubx—stained tissues as described above, being careful to not oversaturate the image in the cells of interest. We then quantified expression for each cell one-by-one in Vaa3D by using circular markers 2 voxels in diameter, taking the mean intensity in that area.

### Fly strains and husbandry

All flies were cultured and kept as adults in plastic vials containing standard cornmeal/molasses medium at 25°C unless otherwise indicated.

Wild-type strains used: Canton S (Bruce Baker, JFRC), Oregon R (Julide Bilen, JFRC), *yw* (Carmen Robinett, JFRC), Hawaii-07 (Drosophila Species Stock Center, UCSD 14021–0231.54), and Connecticut-07 (Drosophila Species Stock Center, UCSD 14021–0231.56).

Twin-spot MARCM [10]: *hs-FLP; FRT40A*, *UAS-CD2*::*RFP*, *UAS-GFP-miR* and *FRT40A*, *UAS-CD8*::*GFP*, *UAS-CD2-miR; R24B02-GAL4(attP2)*

Ubx expression: *yw; UAS-Ubx*.*Ia*.*C/TM6b* [34]

Reference line for examining hemilineage 12A: *R24B02-GAL4(attP2)* expression rapidly diminishes in the 12A neurons during metamorphosis [27], so we used it in combination with *10XUAS-IVS-myr*::*GFP-P10(attP40)*, whose mRNA contains an element increasing its perdurance [35], to observe 12A neurons as late as 72 hours after puparium formation (APF).

Introgression of *R24B02-GAL4(attP40)* and *10XUAS-IVS-myr*::*GFP-p10(attP2)* into the Hawaii-07 and Connecticut-07 strains was performed by first crossing *w; R24B02-GAL4(atP40); 10XUAS-IVS-myr*::*GFP-p10(attP2)* to females of the target strain. 20 females expressing GFP in the third instar larval CNS were backcrossed to males of the target strain. This process was repeated for an additional 8 generations. Males and females (~50 each) that expressed GFP were then crossed, and the brightest progeny of the subsequent cross were used to establish a stock. In both introgression strains, this resulted in a stock homozygous for one transgene and heterozygous for the other, inferred from the fraction of GFP-expressing larvae observed in outcrosses. Despite the heterozygous condition of one of the transgenes, the stocks remained stable in large plastic culture bottles with no additional selection for the duration of this study.

### Creation of *LexAop2-myr*::*tdTomato-P10*

We created *LexAop2-myr*::*tdTomato-P10(attP40)* to examine lineage 11 in relation to hemilineage 12A. A fragment containing the sequence for tdTomato was excised from pJFRC22 [35] by BamHI/XbaI digestion, then ligated into the BamHI/XbaI digested backbone of pJFRC59 [35]. A transgenic fly line was then generated (Genetic Services, Inc., Cambridge, MA) via phiC31-integrase mediated insertion into *attP40*.

### Induction of Twin-spot MARCM clones

Crosses of 5 males with 5–8 females were set in vials containing dry-yeast supplemented cornmeal/molasses food. Crosses were subsequently transferred to fresh vials every 24 hours for 4 days. Vials were heat shocked in a 37°C water bath for 1 hour, then immediately returned to 25°C. At various time points following heat shocks, white puparia were collected from the vials. Thus, our clone inductions are timed as hours before puparium formation (BPF) for the sake of temporal precision, but we report clone induction using the equivalent hours after egg laying (AEL) for convention. We use 120 hours as the interval between egg laying and pupariation at 25°C. Puparia were dissected either immediately or after a further 24 hours of 25°C incubation. Single neuron clones examined at 24 APF were sorted into classes if they shared distinctive morphological features with at least two other examples. Clones were excluded if they represented unique examples or were impossible to trace due to interference from other neurons (*e*.*g*., clones originating from other segments). We excluded 27 such clones.

### Rearing conditions for testing wing behaviors

Crosses were set at the indicated temperature at 60% relative humidity with 4–5 males and 5–8 females per vial, 4–8 vials for each genotype/condition. Morphological data were pooled from multiple biological replicates, each of which includes new parent flies on a new batch of food.

### Cylinder drop assay

Adults were sorted into groups of 10 males and 10 females per vial under cold anesthesia and aged for 3–6 days at 21.8°C on a 16h:8h light/dark cycle. Flies were tested between ZT12 and ZT15 using a method modified from Benzer [36]. For each condition, three vials of flies were sequentially tapped into a funnel inserted into a 1-L graduated cylinder (63mm diameter) lined with strips of flypaper (Victor^®^ Super Fly Roll M521). The flypaper was photographed using an Apple iPhone5s. Images were analyzed using a custom MATLAB script to determine for each fly the height at which it contacted the flypaper.

### Tests of courtship song

Adult males were collected within 18 hours after eclosion and individually housed at 21.8°C for 2–4 days prior to testing. Courtship song tests and recordings were performed as described by Shirangi et al. [37] and Arthur et al. [38].

### Tests of spontaneous and evoked flight

Late-stage pupae were transferred to a 21.8°C incubator until adults emerged. Adults were collected and acclimated to the new temperature and 16h:8h light cycle for 3 days prior to testing. Flies were individually released onto a 5mm x 5mm platform surrounded by a moat of water and covered by a white dome, creating an environment such that the only mode of escape involves flight. Flies were recorded by high-speed video cameras to record the time it took to initiate flight. For the spontaneous flight assay, we recorded the time it took for each fly to spontaneously leave the platform, up to one minute. For the evoked flight assay, an artificial looming stimulus was projected onto the dome once the fly appeared on the platform. This stimulus was a black disc that increased in diameter at a constant velocity: an initial disc of 10° increased to 180° at an *r*/*v* of 40 and a rate of 360Hz as described by von Reyn et al. [39].

### Statistical analyses

Except where otherwise indicated, R 2.15.2 (R Core Team, 2012) was used for all statistical tests, in the simulation, and in the creation of several figures, using base functions in combination with the following packages: “fmsb” (Minato Nakazawa), “binom” (Sundar Dorai-Raj), and “ggplot2” (Hadley Wickham).

## Results

### Typical morphology and development of hemilineage 12A

**Fig 2:**
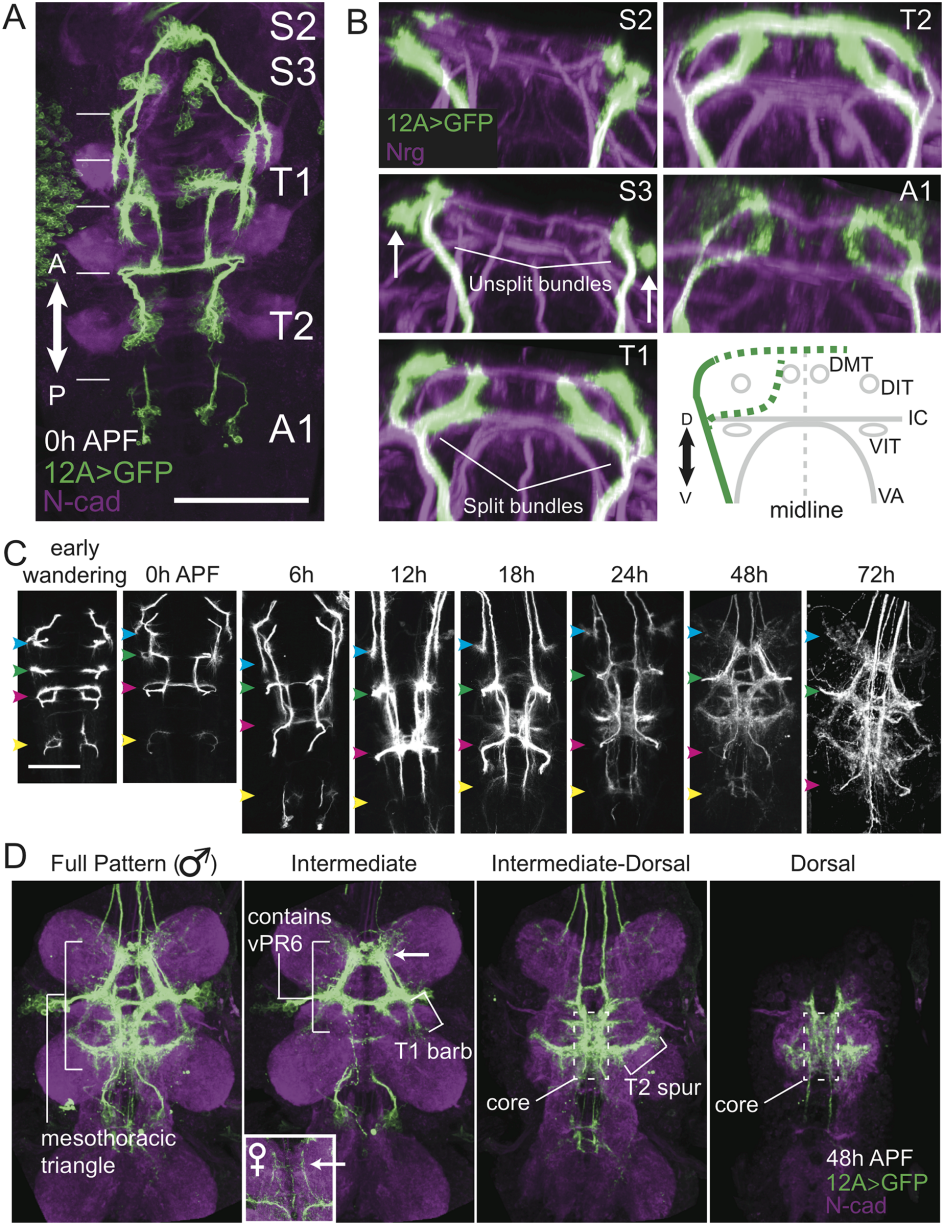
Typical development of hemilineage 12A. (**A**) Confocal z-projection (dorsal view) of the ventral CNS showing the hemilineage 12A clusters at puparium formation (0 hr APF). Parts of the brain have been digitally removed to allow for an unobstructed view of S2 and S3. Segment labels are aligned with the cluster of cell bodies that correspond with that segment. Note that the actual position of the neurites in the dorsal neuropil (indicated by thin, white horizontal lines on the left) are offset from the cell bodies. N-cad: anti-DN-cadherin. Scale bar 75 μm. (**B**) Transverse optical sections of segments S2-T2. Arrows in S3 point to descending neurite bundles from S2. Nrg: anti-Neuroglian. The schematic shows the relation of 12A neurites to key landmarks. The solid green line indicates the invariant portion of the bundle across segments. The dashed green lines indicate the parts of the neurite bundle that vary between segments. Ellipses indicate longitudinal tracts stained by anti-Neuroglian that we used as landmarks. DMT: dorso-medial tract, DIT: dorso-intermediate tract, VIT: ventro-intermediate tract, IC: intermediate commissure, VA: ventral arch. (**C**) Confocal z-projections of 12A neurons at various developmental time points. Only a dorsal substack of the VNC is shown for clarity. myr::GFP expression was very low by 72 hours APF (gain adjusted in example). Colored arrowheads indicated corresponding points of dorsal neuropil: blue indicates terminal branches from S2 neurons; green (T1), magenta (T2), and yellow (A1) arrowheads indicate the points at which neurons from the corresponding segments project into the dorsal neuropil. All images are to scale. Scale bar 50 μm. (**D**) Various landmarks in the 12A pattern at 48 hours APF, which is identical to the adult pattern [27]. The full pattern in a male is shown on the left, followed by three substacks to highlight specific features. The arrow indicates the projections of male specific vPR6 neurons and the corresponding location in females (inset). Genotype is *w; R24B02-GAL4(attP40); 10XUAS-IVS-myr*::*GFP-p10(attP2)*.

We first established the typical morphology of the hemilineage 12A neurons, defined as the phenotype most common across genetic and environmental conditions. 12A is present in segments S2 (maxillary) through A1 (Fig 2A and 2B), although the 12A neurons in T3 undergo programmed cell death soon after puparium formation. In all segments, 12A projects a neurite bundle to the dorsal-most region of the neuropil via a route lateral to the dorso- and ventrointermediate longitudinal tracts (DIT and VIT; Fig 2B); in T2 this dorsal bundle continues around the neuropil to project contralaterally. The primary bundles in T1, T2, and A1 all typically split to produce an additional intermediate bundle that first runs between the DIT and VIT, then turns dorsally.

Fig 2C shows the developmental progression 12A during metamorphosis. The immature 12A neurites are restricted to their segment of origin through the feeding period but resume growth around the transition to the wandering period, eventually forming intersegmental projections. Major growth continues until about 48 hours after puparium formation (APF; Fig 2C and 2D), at which point the 12A neurons exhibit adult-like morphology [27]. The S2 neurites project to the anterior edge of the T1 neuromere and are confined to that region. The 12A neurites from the S3, T1, and T2 segments converge onto a dorsal region of a complex neuropil (the tectulum)[40] that is largely centered in T2. They form a pattern that has been described as the mesothoracic triangle, which is implicated in courtship song production (Fig 2D) [41].

The mesothoracic triangle has several landmarks that relate to the 12A neurons (Fig 2D). The anterior apex of the triangle contains few neurites in females, but many neurites in males (Fig 2D, inset). This sexually dimorphic structure is composed of the vPR6 neurons that are involved in courtship song [27,41]. The lateral base corners of the triangle are made up of neurites descending from T1 and ascending from T2. We call the anterior aspect of the lateral corner the “T1 barb” because it contains mainly neurites from T1, and the posterior aspect the “T2 spur”, because it contains mainly neurites from T2. The “core” of the triangle contains neurites from S3, T1, and T2, and extends from the level of the intermediate commissure to the dorsal-most aspect of the neuropil.

### The 12A neurite bundles in T2 exhibit highly variable morphology

**Fig 3:**
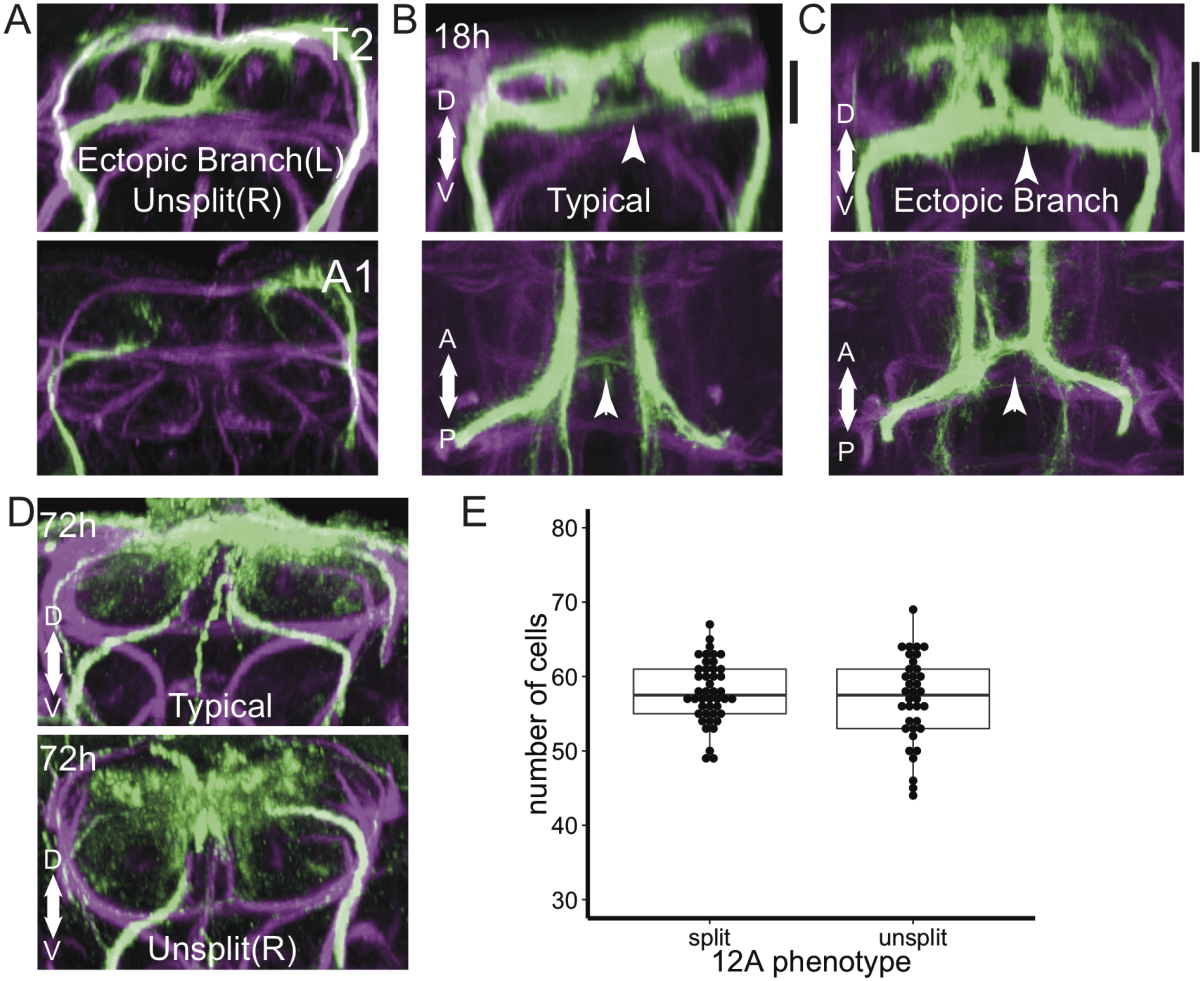
Developmental variability in hemilineage 12A. (**A**) Transverse optical sections showing examples of variation in T2 and A1. Note in T2 the unsplit bundle of the hemilineage on the right and the ectopic branch on the left. (**B-C**) Two examples of T2 at 18h APF. Upper panels are transverse optical sections, lower panels are partial z-projections corresponding to the depth indicated by black bars in the upper panels. Arrowheads show the location of ectopic branches in C and the corresponding location in B. (**D**) Transverse optical sections of two examples of T2 at 72h APF. The upper panel shows an animal with typical branch morphology, whereas the bottom panel shows an unsplit bundle in the right hemisegment. (**E**) comparison between number of cells in split hemilineages (n = 44 hemilineages) and unsplit hemilineages (n = 36 hemilineages) in late wandering third instar larvae. We did not observe a significant difference in cell number (p = 0.36 Welch’s t-test), indicating the difference between the split and unsplit phenotype is not due to differences in neuronal proliferation or death.

The splitting of the 12A bundle into dorsal and intermediate bundles in T1 occurred in all but one of the 1,454 examples in this study. In T2, by contrast, the 12A bundle often failed to split, so only the dorsal bundle was evident (Fig 3A). This variation was highly discontinuous in that we rarely observed intermediate phenotypes (*i*.*e*., weak intermediate bundles). The frequency of unsplit hemilineages ranged from 4–50% depending on genetic background and rearing temperature; the effects of these factors are discussed in more detail below.

In addition to the bundle splitting variation, we also saw higher order variation in the presence of ectopic branches (Fig 3A). In cases in which the 12A bundle split, the intermediate bundle often branched at the level of the intermediate commissure, with one bundle continuing along the typical route and an ectopic branch continuing along the intermediate commissure to the midline. From there, it either crossed the midline or turned dorsally. Ectopic branching was observed in T1 at low frequencies under most conditions (0–6%), but was more common in T2 (6–73%).

Both the unsplit variant and ectopic branch variant reflect developmental variation that has consequences for adult neuronal morphology. We found examples of unsplit hemilineages as late as 72 hours APF (Fig 3D). Evidence for the ectopic branch was ambiguous at late timepoints because of the increasing complexity of the 12A pattern and global shifts in major tracts and commissures. However, examples could be identified in 18 hour APF animals (Fig 3C), and these ectopic projections either contributed to an intermediate level commissure or projected dorsally between the DMTs. Notably, the intermediate commissure targeted by ectopic branches varied in GFP intensity between animals (Fig 3B and 3C). We did not observe any gross changes in the mesothoracic triangle that correlated with either phenotype.

12A variability is not due to toxicity of specific transgenes, because we observe it regardless of expression system (GAL4 or LexA) or fluorescent reporter (myr::GFP, myr::TDTomato, CD8::GFP, CD2::RFP, and others). Moreover, variability is mostly restricted to T2, even though the transgenes are expressed at uniform levels across segments. We also found no difference between the number of labeled cells in split hemilineages versus in unsplit hemilineages (Fig 3E). We conclude that variable splitting and ectopic branching are due to variability in either pathfinding or cell fate determination.

### Late-born classes of 12A neurons in T2 exhibit variable pathfinding

To better understand the population-level 12A phenotypes, we examined individual neurons within the population by using the Twin-spot MARCM technique [10] to induce both single neuron and neuroblast clones at multiple time points between 24 and 120 hours after egg lay (AEL). These clones identify cells born around the time of clone induction and were examined at either puparium formation or 24 hours APF.

**Fig 4:**
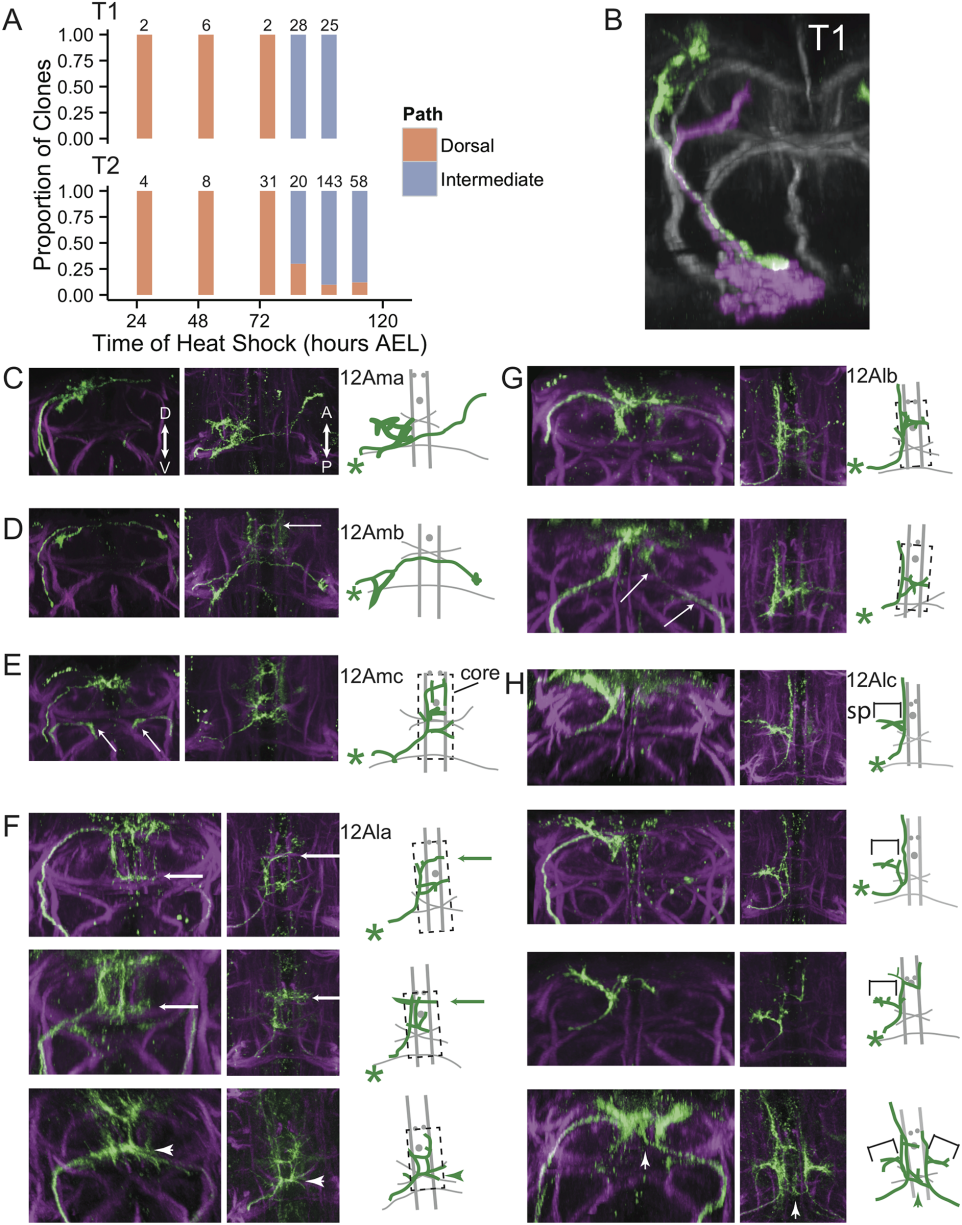
Single-neuron clones reveal variable development of late-born 12A neurons in T2. (**A**) Proportions of single-neuron clones taking either a dorsal or intermediate path in segments T1 or T2. Each bar shows the proportion of clones recovered from heat shocks at the time point on the x-axis. Numbers of clones are indicated above each bar. (**B**) Transverse optical section of T1 showing a twin-spot MARCM clone at 0 hours APF. Green and magenta show a single-neuron and neuroblast clone from the same neuroblast. The magenta neurons are all born after the green neuron. Neuroglian-labeled tracts are shown in grayscale. (**C-H**) Single-neuron clones of 12A in T2 at 24h APF. Transverse optical sections are shown on the left, followed by a z-projection of a dorsal substack, then a tracing of the dorsal view. In tracings, green indicates the clone and gray indicates landmarks as seen by anti-Neuroglian staining (magenta). Green asterisks indicate the point most proximal to the cell soma at which the neurite enters the substack. Dashed boxes indicate the core region of the mesothoracic triangle. (**C**) Example of a 12Ama clone induced at 72h AEL. (**D**) Example of a 12Amb clone, induced at 72h AEL. Arrow points to arbor originating from a T1 clone that is not part of the traced clone. (**E**) Example of a 12Amc clone, induced at 72h AEL. Arrows point to unrepressed signal that is not part of the clone. **(F**) Three examples of 12Ala clones. Note that the top clone routes along the dorsal path while the other two route along the intermediate path. While all three clones produce contralateral projections at the level of the intermediate commissure, only the bottom clone does so at the location of the ectopic branch of the neurite bundle (arrowheads). The top two clones produce this branch at a more anterior location (long arrows). (**G**) Two examples of 12Alb clones, induced at 96h AEL. Note the upper clone takes the dorsal path, whereas the lower example takes the intermediate path but otherwise looks identical. Arrows point to unrepressed signal from the right hemilineage and is not part of the clone. (**H**) Four examples of 12Alc clones induced at either 96h or 108h AEL. Projections into the T2 spur (sp) are indicated with brackets. The top two clones take either the intermediate (top) or dorsal (second from the top) path to an intermediate target, after which they look nearly identical and remain ipsilateral. In the third clone, the longitudinal projection jumps across the midline and continues projecting anteriorly. The bottom example shows two 12Alc clones, one on each side. The clone on the right sends a branch across the midline at the same point as the ectopic commissure (arrowheads); compare this example with the full 18h APF patterns in Fig 3C and the 0h APF example in Fig 3A.

Of 327 single neuron clones in either T1 or T2, none showed a branched neurite that traversed both the dorsal and the intermediate bundles, indicating the dorsal and intermediate bundles represent two distinct populations of neurons. In T1, neurons born prior to 74 hr AEL projected their neurite in the dorsal bundle, whereas those born at later times had their neurite in the intermediate bundle (Fig 4A). The transition from one cell type to the other is illustrated by a twin-spot clone induced at 74 hours AEL in which the single neuron had its neurite in the dorsal bundle whereas the later born neurons were all in the intermediate bundle (Fig 4B). The pattern of clones from T2 was consistent with this dorsal-intermediate transition, although 27 of 221 late-born clones projected through the dorsal bundle. As seen below, these 27 dorsal clones were variants of neuronal types that usually project through the intermediate bundle. We conclude that for hemilineage 12A in both T1 and T2, the neuroblast first produces neurons that take a dorsal path and then switches at about 74 hr AEL to producing neurons that take the intermediate path.

The 202 single-neuron clones in T2 that we examined at 24 hours APF further allowed us to subdivide the late-born neurons into subclasses and to relate the unsplit and ectopic branch phenotypes to mature neuronal morphology. We focused on neurons born around the dorsal to intermediate transition (72 hours AEL) and at two time points when only intermediate path neurons should be produced (96 and 108 hours AEL).

Neurons could be sorted into 6 classes (Fig 4 and Table 1). Clones induced at 72 hours AEL were all dorsal-routing and fell into three of these classes. 12Ama neurons (Fig 4C; n = 12) and 12Amb neurons (Fig 4D; n = 3) both projected contralaterally to the dorsolateral neuropil. 12Amc neurons (Fig 4E; n = 13) projected to the dorsal midline, where they exhibited dense branching that extended throughout the dorsal-most part of the core of the mesothoracic triangle.

**Table 1:** Summary of single neuron clones in T2 examined at 24 hours APF.

Timing of clone induction (Hours AEL)	Class	Total	Dorsal	Intermediate	Ectopic Contralateral*
**72 ± 2**	**12Ama**	12	12	0	0
**72 ± 2**	**12Amb**	3	3	0	0
**72 ± 2**	**12Amc**	13	13	0	0
**96 ± 2**	**12Ala**	85	9	76	30
**96 ± 2**	**12Alb**	14	1	13	0
**96 ± 2**	**12Alc**	17	3	14	10
**108 ± 2**	**12Alc**	58	7	51	17

*Number of clones that produce ectopic contralateral projections as in Fig 4F and 4H.

Single neuron clones induced with 96 and 108 hour AEL heat shocks fell into the remaining 3 classes. 12Ala neurons (Fig 4F; n = 85) projected to the core of the mesothoracic triangle like 12Amc, but 12Ala branches occupied a space that extended ventrally to the level of the intermediate commissure. 12Ala neurons exhibited a large degree of fine-scale diversity, and some examples extended branches along the intermediate commissure, corresponding to path of the ectopic branch of the primary neurite bundle. We conclude that the ectopic branch includes branches from 12Ala variants.

12Alb neurons (Fig 4G; n = 14) projected into T1 along a longitudinal path laterally adjacent to the DMT, extending branches medially into the core of the mesothoracic triangle at about the midpoint of the longitudinal projection.

12Alc neurons (Fig 4H; n = 75) projected along the same longitudinal tract as the 12Alb neurons, except they produced lateral branches that became part of the T2 spur of the mesothoracic triangle. In some 12Alc neurons, the longitudinal projection either projected branches across the midline or completely crossed the midline to proceed along the contralateral longitudinal path. While the point at which 12Alc variants crossed the midline varied among clones, they usually crossed at the commissure corresponding to the path of the ectopic neurite branch. We conclude that the ectopic branch also includes branches from 12Alc variants.

Most of the examples of 12Ala, 12Alb and 12Alc neurons were intermediate-routing, as expected given their late induction. However, 20 of the 174 cells were dorsal-routing but still showed the characteristic arborization fields typical of their class (Fig 4F, 4G and 4H). Therefore, despite projecting along the wrong pathway, these cells recovered to make arbors in their appropriate regions. We conclude that the unsplit phenotype occurs when late born classes of neurons route dorsally instead of via their typical intermediate path.

**Fig 5:**
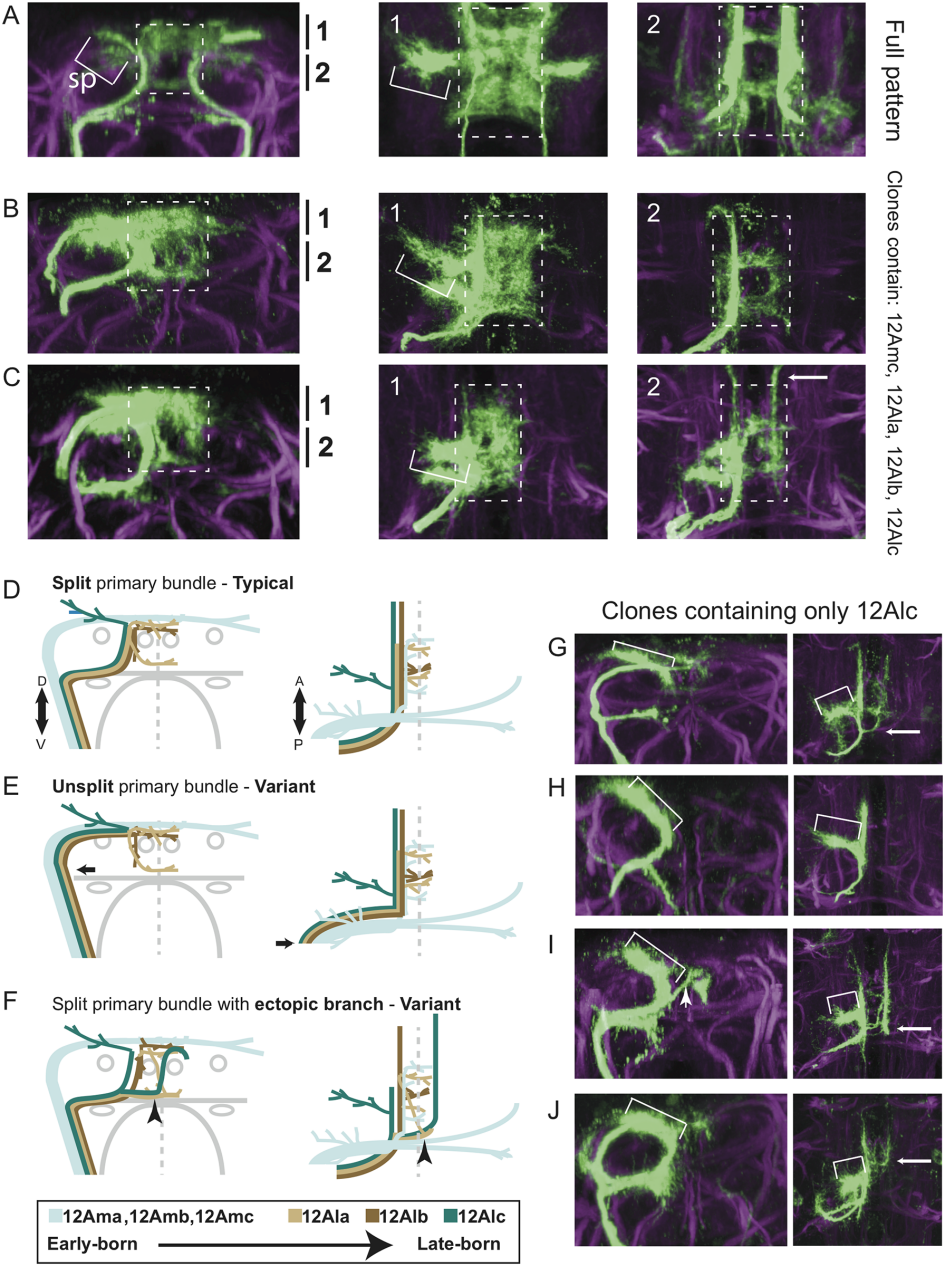
Neuroblast clones further demonstrate 12A variability. (**A**) Full 24B02-GAL4 pattern in T2 at 24h APF. A transverse optical section is shown on the left, with two dorsal views of partial z-projections on the right, representing two levels along the D-V axis (indicated by black bars). The core region is indicated by dashed boxes and the T2 spur (Sp) is indicated with a bracket. (**B-C**) Two neuroblast clones induced at 72h AEL and examined at 24h APF. Note the presence of the contralateral longitudinal projections in C (arrow), but not B. **(D-F)** Schematic of the hemilineage 12A neurons in T2 at 24 hours APF. Neurons are colored according to the key at the bottom of the Fig, with landmarks (as in Fig 2B) shown in gray. Transverse views are shown on the left, dorsal views on the right. Some features of the neurons are slightly shifted or exaggerated as a visual aid. Only one neuron of each class is shown, but each hemilineage contains multiple neurons of each neuron class. This schematic does not capture all possible neuronal classes produced by this hemilineage, only those that we recovered this study. **(D)** Typical development of 12A in T2. **(E)** Atypical unsplit 12A phenotype in which the late-born neurons all take the dorsal path (arrow), although they still reach the appropriate target zone. **(F)** Atypical ectopic branch 12A phenotype in which 12Ala and 12Alc neurons produce branches that split off from the intermediate bundle (arrowhead). 12Alc can also ectopically cross the midline at more anterior locations, but these variants can only be seen with clones and were not quantified in multiple genotypes/temperatures. (**G-J**) Neuroblast clones induced at 96h AEL and examined at 24h APF. z-projections of the full dorsal neuropil are shown. The clones contain only 12Alc neurons. Clones vary with respect to neurons taking either the dorsal (G) or intermediate route (H and I) or both (J). They also vary with respect to contralateral projections (arrows). (**I**) Note the ectopic contralateral projections run long the ectopic branch route (arrowhead). Compare this clone with Figs 3A, 3C and 4H.

Neuroblast clones (Fig 5 and Table 2) supported our conclusions from the single-neuron clones that late-born neuron types can take either the intermediate or dorsal route without major consequences for subsequent steps of development. However, late clones containing only 12Alc neurons provided an additional insight: while 14/15 clones contained neurons that all initially took either the intermediate route (10 clones) or the dorsal route (4 clones), one clone contained both dorsal and intermediate-routing neurons (Fig 5J). This indicates that, at the neuronal population level, mixed phenotypes are possible but infrequent.

**Table 2:** Summary of neuroblast clones in T2 examined at 24 hours APF.

Clone induction (Hours AEL)	Total	Dorsal-only	Intermediate-only	Both	Contralateral Longitudinal*
**72**	4	0	1	3	3
**96**	12	3	8	1	6
**108**	3	1	2	0	1

*Number of NB clones that innervated contralateral longitudinal tract as in Fig 5C, 5G, 5I and 5J.

### 12A pathfinding variability is cell-intrinsic and induced by Ultrabithorax

**Fig 6:**
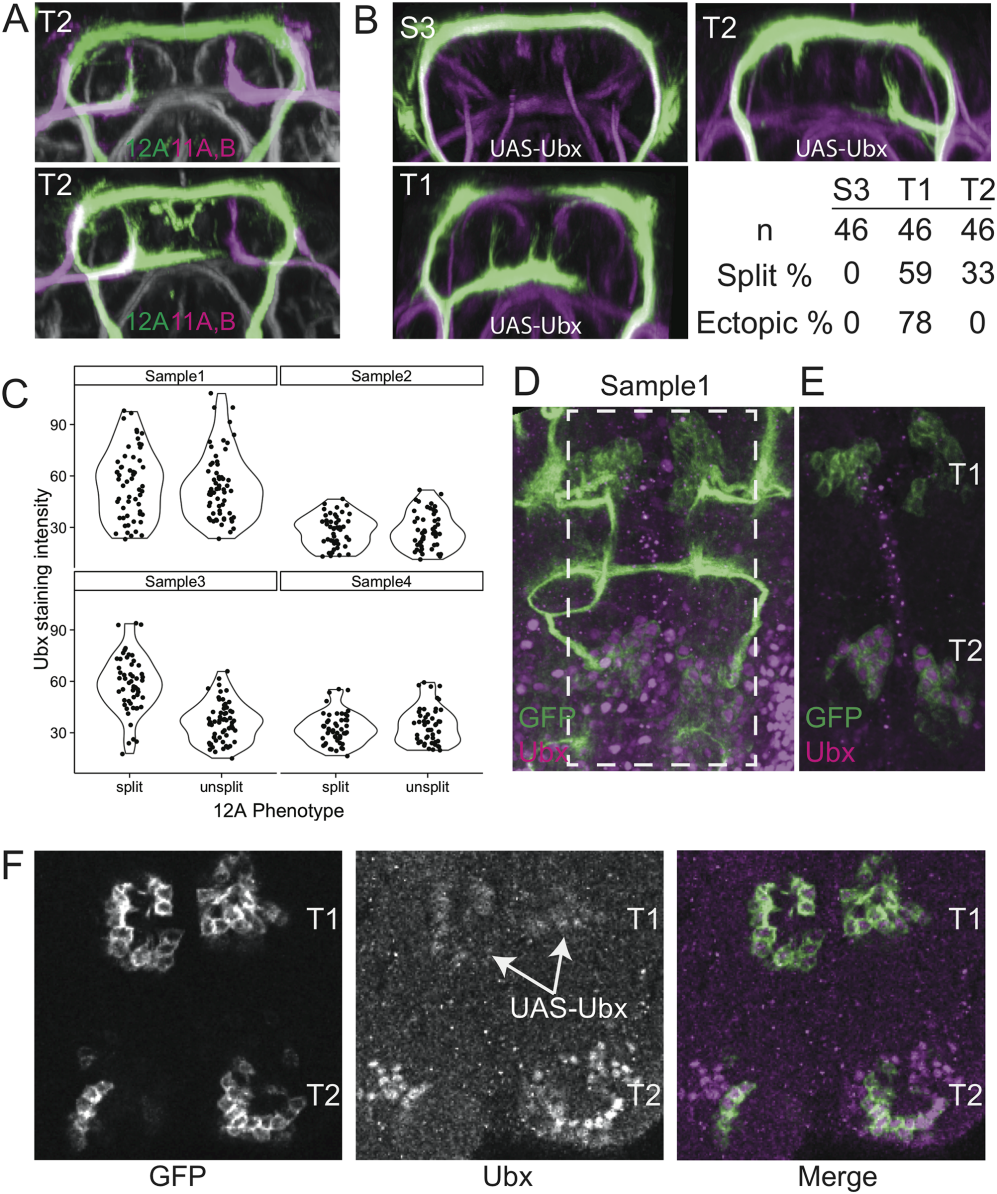
12A in T2 is a variability “hot-spot.” (**A**) Two examples of transverse projections through the T2 neuromere showing the neurite bundles of hemilineages 11A and 11B (magenta) relative to those of 12A (green). The right 12A hemilineages failed to split but the lineage 11 projections were invariant. Lineages from the genotype: *w; R26B05-LexA(attP40)/LexAop2-myr*::*tdTomato-P10; R24B02-GAL4(attP2)/UAS-myr*::*GFP-P10*. (**B**) Single examples of S3, T1, and T2, showing the effects of expressing UAS-Ubx.Ia. The immature neurite bundles in S3 are consistently transformed toward the unsplit T2 morphology. The 12A neurons in T1 become T2-like with respect to variability in the failure to split and in forming ectopic branches. Percentages shown are for the fraction of hemilineages that split into a dorsal and intermediate bundle and the fraction of split hemilineages that have an ectopic branch. (**C**) Quantification of anti-Ubx staining for four individuals that showed bilateral asymmetry. A difference in staining intensity was only observed in one of the four individuals. (**D**) An image stack from one of the four individuals (Sample1), with GFP in green and anti-Ubx in magenta. (**E**) A single optical section of the sample in D (showing area in the dashed box). Note the Ubx expression in T2 and lack of expression in T1. (**F**) When Ubx was expressed in 12A neurons in T1 using R24B02-GAL4 with UAS-Ubx.1a, Ubx was detectable (arrows) in the T1 neurons but still very low when compared to the endogenous levels in T2.

The variable pathfinding we observe by the late-born 12A neurons in segment T2 could be due to extrinsic variability (*e*.*g*., variable distribution of guidance cues within the VNC), intrinsic variability (*e*.*g*., variable expression levels of guidance receptors in the 12A neurons), or both. We first asked whether other neurons similar to 12A neurons in location and morphology are similarly variable. In T2, hemilineage 11A neurons project their neurite bundle along the 12A intermediate bundle and the 11B neurons project along the 12A dorsal bundle. If 12A variation results from a variable external environment, then 11A neurons might also vary, with an expected correlation between 11A and 12A phenotypes. We examined 11A, 11B, and 12A neurons together in 8 animals, for a total of 16 hemisegments. 12A was unsplit in five of the hemisegments, but 11A and 11B never varied (Fig 6A). We also have not seen any examples of variability in the lineage 11 primary neurite bundles in dozens of examples seen outside of this study (unpublished observations).

In a second approach, we took advantage of the similarity in 12A primary neurite morphology between T1 and T2. The difference in segmental identity between 12A in segments T1 and T2 is dependent on Ultrabithorax (Ubx) [32]: the 12A neurons in T2 express Ubx, whereas the T1 homologs do not. Ubx is thus responsible for developmental differences between the T1 and T2 neurons, but does not affect the location or presence of the intermediate branch in the typical case. We therefore used *R24B02-GAL4* to express the Ubx Ia isoform in all 12A hemilineages, which transforms T1 neurons toward the T2 fate. This allowed us to ask what happens when the T2 neurons develop in a T1 environment.

Ubx expression in the 12A neurons in T1 resulted in variable splitting and ectopic branching, at rates consistent with what is typically seen in T2 (Fig 6B). Interestingly, within animals that now expressed Ubx in all 12A hemilineages, the proportion of variants differed between T1 and T2. T1 had a higher percentage of split bundles (59% Vs 33%; p = 0.02, chi-squared test) and ectopic branches whereas T2 had no ectopic branching (78% Vs 0%; p = 6.5e-06). These differences probably do not reflect a difference in Ubx levels, as we discovered in the following experiments. The key finding, though, is that when a T2 identity was imposed on the 12A neurons in segment T1, they then showed the types and high levels of variation that characterizes their T2 counterparts. This shows that the variability exhibited by the T2 cells is primarily a cell intrinsic property of these cells that can be exhibited in different environments.

Ubx could cause 12A variability in one of two ways. One possibility is that Ubx, at low to moderate levels, establishes an epigenetic state in which developmental gene networks are somewhat unstable, leading to the variable developmental outcome (Model 1: Ubx specifies variability). Alternatively, Ubx could directly specify different 12A phenotypes at different expression levels, and variable Ubx levels lead to the developmental variability (Model 2: Variable Ubx causes variable phenotypes). Two observations lead us to conclude that Model 1 is correct. First, when we examined endogenous Ubx expression in animals that exhibit bilaterally asymmetric 12A phenotypes, we saw no consistent difference in Ubx expression between the split and unsplit hemilineage (Fig 6C, 6D and 6E). Thus if Ubx directly specifies the variable 12A phenotypes, 12A neurons must be exquisitely sensitive to Ubx expression differences. Second, we observe all three phenotypes at both the endogenous Ubx levels in T2 and in the very low levels driven by Ubx in T1 (barely detectable levels of immunoreactivity; Fig 6F). Thus all three phenotypes can be produced by a wide dynamic range of Ubx expression. Considering the inconsistency of these two results under Model 2, we conclude that Model 1 is correct, and that Ubx specifies a cellular state that is developmental variable.

Ubx expression also partially transformed the S3 neurons toward the T2 fate in that the 12A bundles show the T2 feature of projecting dorsally across the midline, but they do not show the bundle splitting characteristic of T1 and T2. The S2 cells, by contrast, were unaffected by Ubx expression. The reduced effect of Ubx expression on the subesophageal lineages is not surprising, because neurons in S2 and S3 express other homeotic genes that establish their anteroposterior identity [31], and the S2 and S3 environments differ from the thoracic neuromeres with respect to neuronal composition [20,31].

### Genotype and environment interact to affect the variability of the 12A hemilineage

Phenotypic variability can arise from genetic and environmental variation and developmental noise. To begin distinguishing between these sources of variation, we first examined the effect of genotype by quantifying phenotype frequencies in various genetic backgrounds.

We first crossed females of a tester line (“T”–*w; R24B02-GAL4; UAS-myr*::*GFP-P10*) to males of Canton S, Oregon R, and *yw* (F1 hybrids hereafter referred to as T/CS, T/OR, and T/*yw*, respectively). To test the possibility that variability is unique to laboratory strains, we used nine rounds of backcrossing to introgress the transgenes into two recently derived strains from Hawaii and Connecticut (hereafter referred to as HI and CT). Finally, we also examined hybrids resulting from Hawaii females crossed to Connecticut males (HI/CT) and vice versa (CT/HI).

**Fig 7:**
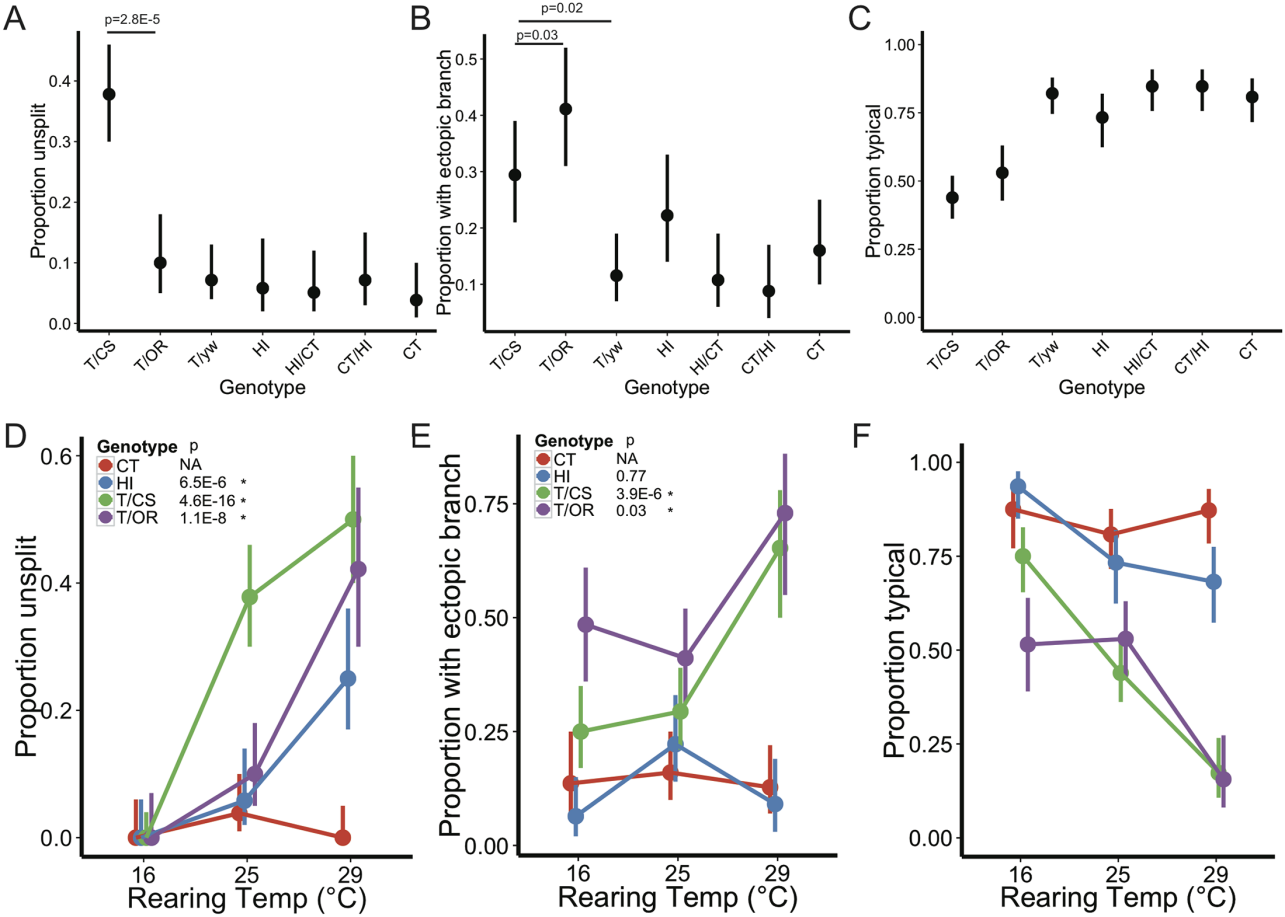
12A variability is sensitive to genetic background and temperature. (**A-B**) Proportions of unsplit hemilineages and ectopic branching in various genotypes at 25°C. (**A**) T/CS proportions were significantly different from all other groups (p < 0.05), but only the comparison with T/OR is shown. (**B**) T/OR had the largest proportion of ectopic branches, followed by T/CS. (**C**) Proportion of hemilineages with the typical morphology. (**D-E**) Effect of temperature on bundle splitting and ectopic branching. Data at 25°C are from 7A and 7B. p values are for comparisons between 16°C and 29°C for each genotype. (**F**) Effect of temperature on typical morphology. For all panels, vertical error bars represent 95% confidence intervals. Chi-squared tests were used to obtain p values and 95% CI. Holm correction for multiple comparisons (21 pairwise comparisons) was applied to p values in Fig 7A and 7B. T/CS: progeny of tester strains crossed to Canton S; T/OR: tester/Oregon R; T/yw: tester/*yw*; HI: Recently derived strain from Hawaii; CT: Recently derived strain from Connecticut.

At 25°C, we observed variation in all seven genotypes, including the wild-derived hybrid strains, HI/CT and CT/HI (Fig 7A–7C). Most genotypes exhibited failure of the hemilineage to split into dorsal and intermediate bundles at rates between 4–10%, but T/CS stood out with 38% of hemilineages failing to split. T/OR exhibited the most ectopic branching at 41% of total split hemilineages, followed by T/CS with 29%. We conclude that 12A variation in T2 is present in both laboratory and wild-derived strains but that different strains exhibit distinct degrees of variability.

The fact that we typically saw bilateral asymmetry suggested that 12A development is highly influenced by non-genetic sources of variation. To test the effect of an environmental condition, rearing temperature, on 12A variation, we examined 12A morphology in four genotypes (T/CS, T/OR, HI, and CT) at two additional rearing temperatures, 16°C and 29°C. Surprisingly, all of the four genotypes showed normal bundle splitting when reared at 16°C (Fig 7D) but after rearing larvae at 29°C, the T/CS, T/OR, and HI strains showed substantial suppression of bundle splitting. Only the CT strain remained stable across all three temperatures.

The response of ectopic branching to elevated temperature differed between the two established lab strains and the two recently established strains (Fig 7E). The HI and CT strains had lower frequencies of ectopic branches and were stable across all temperatures. By contrast, the T/CS and T/OR genotypes had higher levels of ectopic branches that changed with rearing temperature, being stable across 16°C and 25°C but then increasing at 29°C.

When considering the total frequency of atypical 12A phenotypes, the laboratory genotypes had both a greater frequency of atypical morphologies across all temperatures and higher sensitivity to increased rearing temperature than the recently derived genotypes (Fig 7F). We conclude that 12A development is more robust in recently derived strains than in laboratory strains.

Finally, the importance of developmental noise for 12A variation was clear from the surprisingly high frequency of bilateral asymmetry we found for both bundle splitting and ectopic branching (Figs 3A and6A; Table 3). To test whether the alterations in the left and right hemilineages varied independently, we calculated for both bundle splitting and ectopic branching the expected number of symmetrical hemilineage pairs based on our measured frequencies, with the assumption of left-right independence (Table 3). Rates of asymmetry with respect to the bundle splitting phenotype were very close to predictions, with only T/CS animals reared at 29°C showing a small, but statistically significant bias toward symmetry. However, for the ectopic branch phenotype, there were fewer asymmetric animals than expected across all conditions. Hence, the bundle splitting phenotype shows complete left/right independence, while the ectopic branching phenotype is only partially left/right independent.

**Table 3:** Expected and observed frequencies of bilateral asymmetry.

		bundle splitting asymmetry				ectopic branch asymmetry			
Rearing Temp	Genotype	n	proportion	expected proportion	p value	n*	proportion	expected proportion	p value
**16**	**CT**	36	0	0	*NA*	36	0.08	0.24	***1*.*80E-02***
**16**	**HI**	39	0	0	*NA*	39	0.03	0.12	***4*.*30E-02***
**16**	**T/CS**	52	0	0	*NA*	52	0.15	0.38	***4*.*30E-04***
**16**	**T/OR**	33	0	0	*NA*	33	0.06	0.5	***6*.*70E-08***
**25**	**CT**	52	0.08	0.07	0.66	48	0.04	0.27	***5*.*10E-05***
**25**	**HI**	43	0.12	0.11	0.67	38	0.05	0.35	***2*.*20E-05***
**25**	**T/CS**	82	0.46	0.47	0.5	32	0.25	0.42	***4*.*00E-02***
**25**	**T/OR**	50	0.2	0.18	0.72	40	0.25	0.48	***2*.*10E-03***
**25**	**T/*yw***	70	0.14	0.13	0.68	60	0.12	0.2	***1*.*10E-02***
**25**	**HI/CT**	49	0.1	0.1	0.66	44	0.02	0.19	***9*.*70E-04***
**25**	**CT/HI**	49	0.1	0.13	0.35	43	0.02	0.16	***5*.*00E-03***
**29**	**CT**	47	0	0	*NA*	47	0	0.22	***7*.*20E-06***
**29**	**HI**	44	0.32	0.38	0.27	26	0.04	0.17	5.60E-02
**29**	**T/CS**	49	0.35	0.5	***0*.*02***	17	0.12	0.45	***3*.*80E-03***
**29**	**T/OR**	32	0.41	0.49	0.23	12	0	0.39	***2*.*40E-03***

Bold and italic indicates p<0.05; one-tailed binomial test.

*ectopic branch symmetry could only be determined for animals in which both T2 hemilineages split to produce the intermediate bundle.

### Ectopic branching of 12A neurons correlates with delayed flight initiation

We next asked if variation in the development of the 12A interneurons correlated with altered behavioral performance of the flies. We reasoned that the unsplit variants were unlikely to have disrupted circuit architecture because their axons reach their appropriate target sites despite taking an alternate path (Fig 5E). By contrast, the ectopic branch variants must have altered circuit architecture, because neurons that typically exhibit only ipsilateral input and outputs instead produce contralateral projections, depriving the ipsilateral side of some synaptic connections and potentially producing ectopic synapses on the contralateral side (Fig 5F). Unfortunately, the R24B02 driver stops expressing in these neurons before adult emergence so we could not test flies behaviorally and then look at the anatomy of these interneurons in the tested flies. Consequently, we took a statistical approach to compare variation in development to that in behavior.

We approached our behavioral analysis with the assumption that for genotype/temperature conditions in which there is a low frequency of ectopic branching, quantification of a behavior across many individuals will produce a unimodal distribution. If ectopic branching produced a large effect for that behavior, then we expected to see a prominent second mode to appear in conditions that promoted frequent ectopic branching (S1 Fig). Because 12A anatomy is suggestive of a role in wing control [20], activation of 12A neurons in the adult produces wing movements [27], and a subset of 12A neurons are involved in courtship song production [41], we chose to focus on wing-related behaviors: flight (specifically recovery from freefall and voluntary flight initiation), courtship song, and escape.

**Fig 8:**
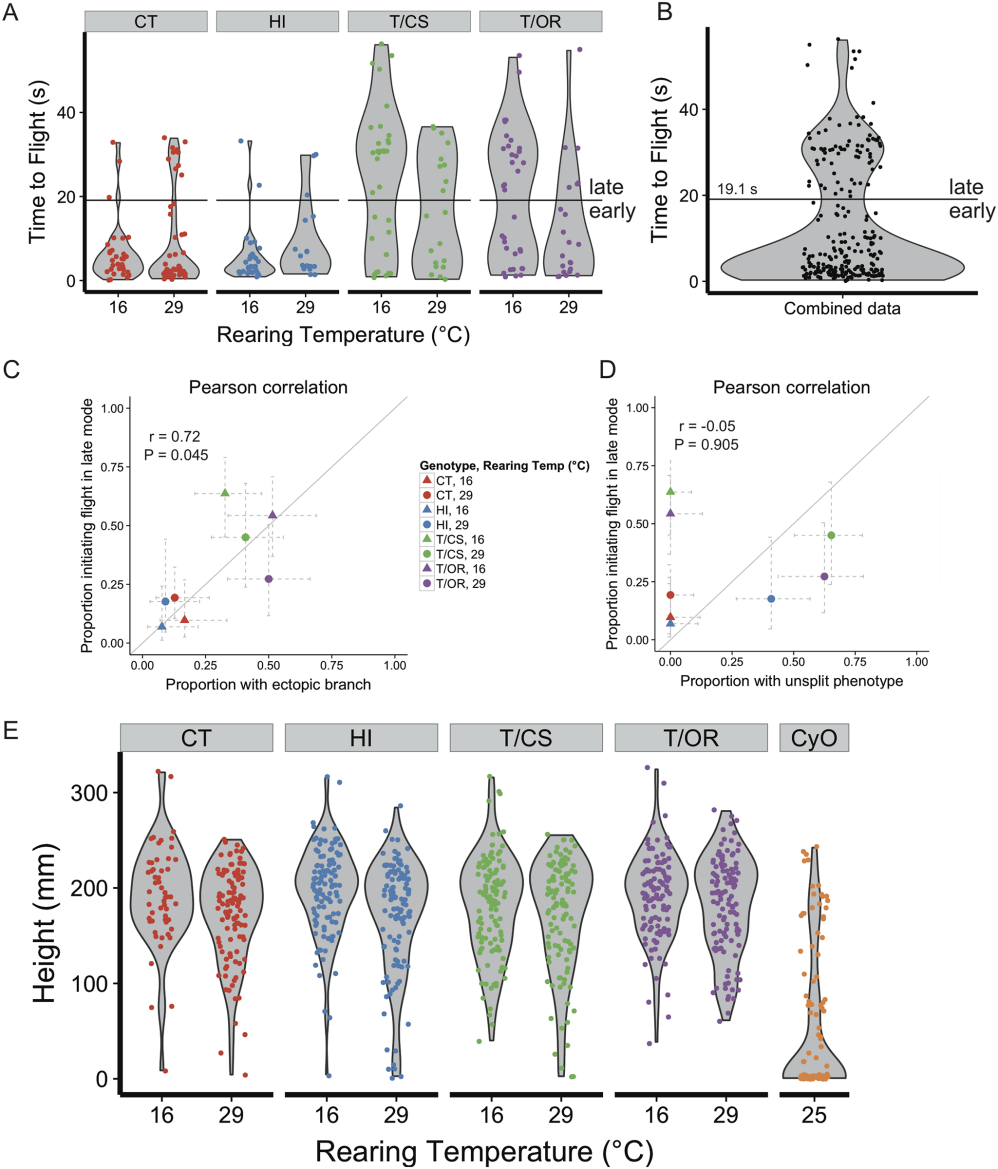
Correlation between ectopic branch phenotype and delayed flight initiation. (**A**) Plot of the time it takes for an individual fly to spontaneously initiate flight after being released onto a small pedestal. The kernel density estimate for each condition was calculated in R using the base density function with the default bandwidth; this is shown as a gray violin plot behind the data points. The spread of data in the T/OR and T/CS groups is much broader than in the CT and HI groups, and bimodality is suggested in the CT (29°C), T/OR (16°C), and T/CS groups. (**B**) Combined data from 8A. Using the kernel density estimate of the combined data, a cutoff was established at the local minimum between the two modes: 19.1 s. This cutoff was then applied to the data in 8A to sort individual flies into early or late groups. (**C**) Pearson correlation comparing the proportion of flies taking off in the late mode to the expected proportion of flies that should harbor the ectopic branch phenotype in either 12A hemilineage in T2. Gray diagonal line indicates the expected 1:1 correlation. (**D**) Pearson correlation comparing the proportion of flies taking off in the late mode to the expected proportion of flies that should harbor the unsplit phenotype (**E**) Ability of flies to recover from free fall. Each point represents the height at which a falling fly hits the side of the test cylinder. Flies that fly poorly (CyO) mostly reach the bottom of the cylinder. Note that the distributions of T/OR (~50% of animals have ectopic branch phenotype) as compared to HI (~8% of animals have ectopic branch phenotype) are not suggestive of a mixed population as with spontaneous flight initation in 8A. Strains as in Fig 7.

We approached our statistical analysis in two steps. We first visually inspected kernel density estimates of data pooled across all conditions for each behavior to determine whether there was a clear multimodal structure to the data. Only spontaneous flight initiation, in which flies were sequentially released onto a small pedestal and allowed to initiate flight voluntarily, showed such a structure (Fig 8A and 8B).

For the second step in our analysis, we first established a cutoff with which to sort individual flies into two groups: early takeoff and late takeoff (Fig 8B). We next tested whether the proportions of early vs. late data points in each condition correlated with the penetrance of the ectopic branching phenotype in that condition. We found that there was a strong correlation between late flight initiation and a high frequency of ectopic branching (Fig 8C). To ensure the robustness of this correlation, we examined a second cohort that was a full biological replicate, and again observed this correlation (S2 Fig). By contrast, the time of flight initiation was not correlated with whether or not the 12A bundle showed the unsplit phenotype (Fig 8D).

Because none of the other behaviors produced clear bimodal distributions with which to perform this correlational analysis (i.e. they did not meet the first criterion of our analysis) (S3 Fig), correlations were not calculated. For example, the ability of flies to recover from freefall showed no discernable difference in the shape of the data distribution between conditions in which half carry the ectopic branch phenotype (*e*.*g*. T/OR) and conditions in which few carry the phenotype (*e*.*g*. HI) (Fig 8E). This does not mean that there was no effect of 12A variability on this behavior, but any potential effect was too small to be detected in a mixed population.

### Other types of developmental variants

We examined 727 animals in this study, or 1,454 examples of each segmental variant of hemilineage 12A (7,270 total hemilineages across all segments, not counting the cells in T3 that undergo programmed cell death). This gave us the opportunity to identify additional uncommon, but naturally occurring, developmental variants that would ordinarily be missed or ignored in focused mechanistic studies.

**Fig 9:**
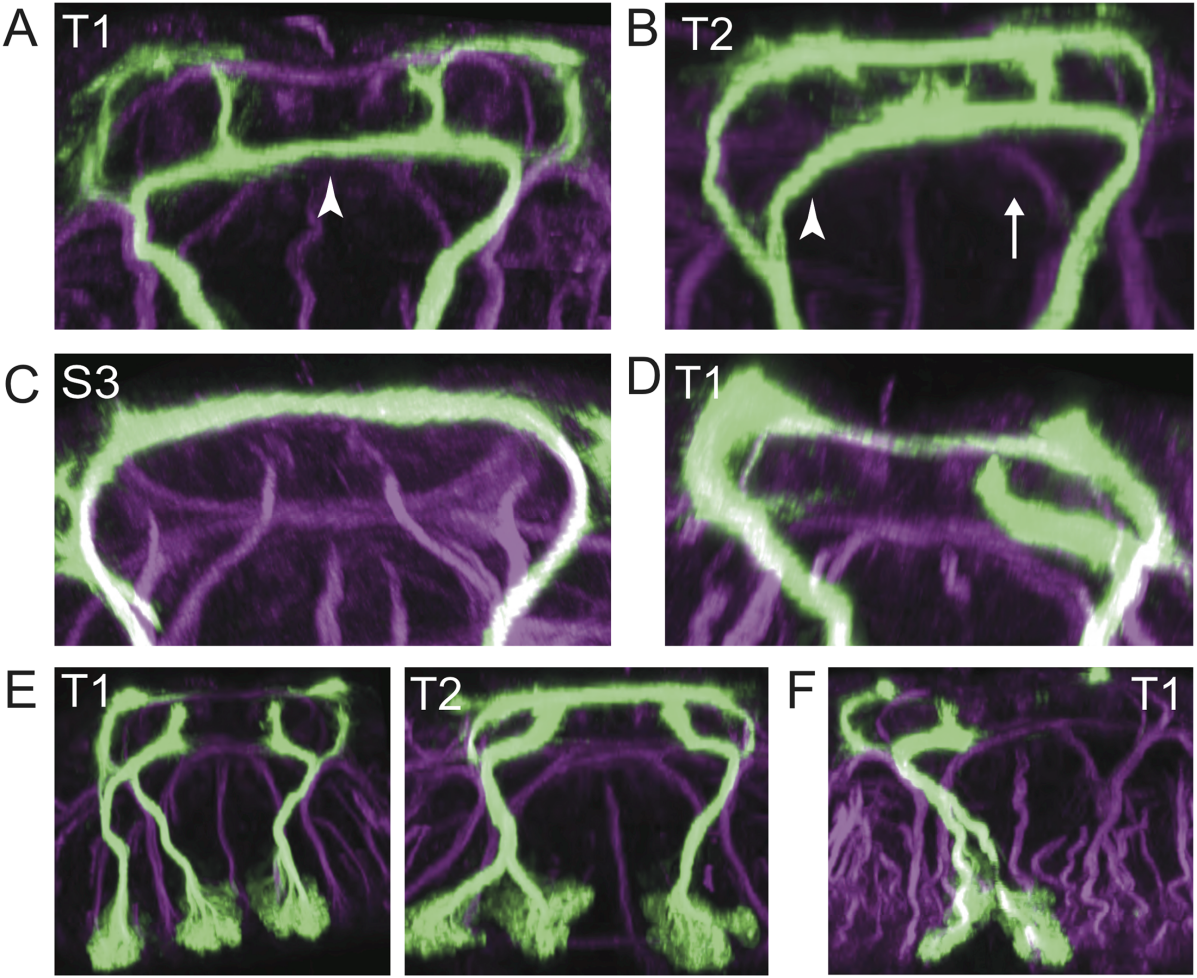
Other examples of 12A variants. (**A**) An example of the ectopic branch phenotype in T1 (compare with the typical T1 in Fig 2B and the ectopic branch phenotype of T2 in Fig 3A). All examples were bilaterally symmetrical with a fully formed commissure (arrowhead), in contrast to 12A in T2, which often exhibits bilateral asymmetry. (**B**) Example of ventral arch routing of the late-born 12A neurons in T2. Compare the branch on the left (arrowhead) with the unoccupied Neuroglian-stained tract on the right (arrow). (**C**) Transverse optical section of S3 in which 12A shows the T2 morphology (with unsplit bundles). Compare with Fig 6B and the variants of 12A that are missing the intermediate bundle as shown in Figs 3A and 6A. (**D**) Transverse optical section of T1 in which the left bundle fails to split. (**E**) Two examples of animals with duplicated hemilineages. (**F**) One of two cases in which one hemilineage ectopically projected all of its neurites to the contralateral side, leaving one T1 hemisegment completely uninnervated by 12A and the other double innervated.

The most common of the additional variants was a new commissural connection in T1 (Fig 9A and Table 4), which was infrequently observed (~0–3% of animals) in most genotype/temperature combinations, but present in ~25% of HI animals and at 12–13% in the HI/CT and CT/HI hybrids. We do not yet know the fate of this novel connection in the HI flies. We think its basis differs from the ectopic branches we see in T2, because the T1 connection is very stereotyped, always symmetrical, and is unresponsive to temperature.

**Table 4:** Summary of uncommon phenotypes.

Rearing Temp (°C)	Genotype	Ectopic Commissure in T1	Premature branch in T2	Duplicated hemilineage
**16**	**CT**	**1 (36)**	0 (72)	0 (360)
**16**	**HI**	12 (39)*	**1 (78)**	0 (390)
**16**	**T/CS**	0 (52)	0 (104)	7 (520)**
**16**	**T/OR**	**1 (33)**	0 (66)	0 (330)
**25**	**CT**	**1 (52)**	0 (104)	0 (520)
**25**	**HI**	10 (43)*	0 (86)	0 (430)
**25**	**T/CS**	**2 (82)**	0 (164)	1 (820)**
**25**	**T/OR**	**1 (50)**	**2 (100)**	0 (500)
**25**	**T/*yw***	**1 (70)**	**2 (140)**	0 (700)
**25**	**HI/CT**	6 (49)*	**1 (98)**	0 (490)
**25**	**CT/HI**	7 (49)*	0 (98)	**1 (490)**
**29**	**CT**	**3 (47)**	**2 (94)**	0 (470)
**29**	**HI**	10 (44)*	0 (88)	0 (440)
**29**	**T/CS**	0 (49)	**1 (98)**	1 (490)**
**29**	**T/OR**	**1 (32)**	0 (64)	0 (320)

All nonzero cells are bolded. Numbers in parentheses indicate total possible examples: the ectopic 12A[T1] commissure was always bilaterally symmetric and thus the number of animals (n) is given, the premature T2 branch was scored on a hemilineage basis (2n), and the number for duplicated hemilineages considers all segmental iterations of 12A (20n).

*Animals homozygous or heterozygous for the HI strain had an elevated frequency of ectopic 12A[T1] commissures.

**9/10 duplicated hemilineages were from T/CS animals, with 7 occuring at 16°C.

We found 9 examples in T2 of 12A variants in which neurons prematurely turn medially at the level of the ventral arch, but still reach either the ipsilateral or contralateral longitudinal tract in which the late born neurons project (Fig 9B and Table 4). The ventral arch is the typical path of the 12B neurons, so it is possible for 12A cells to ectopically exhibit a characteristic of their 12B siblings (the initial neurite path) while retaining other features specific to 12A (targeting the longitudinal tract lateral to the DMT).

The most surprising rare variant was hemilineage duplication (Fig 9E and Table 4). We observed 10 instances (across 6 animals) of two 12A hemilineages in a single hemisegment; these instances were found in multiple segments and not restricted to T2. In these cases, we were able to identify the neuroglian-labeled neurite bundles from all of the other lineages, showing that the duplicated hemilineages came from an ectopic lineage 12 neuroblast, rather than from the change of fate of another neuroblast. The initial neurite bundles of duplicated hemilineages are separate as they leave their clusters, but join together at or within the neuropil. Regardless of the doubling of neuron number, there are no gross morphological defects in either the 12A hemlineages or the other neuroglian tracts in early pupae, although we do not know what happens during subsequent stages of development. Given that 7 of these 10 examples occurred in T/CS animals at 16°C, it is probable that the T/CS genotype is prone to neuroblast duplications when embryogenesis and larval growth occurs at lower temperatures.

Finally, we also observed one case in which the primary neurite bundle of 12A in S3 had the unsplit T2 morphology (phenocopy of Ubx expression; Fig 9C), one case in T1 in which the 12A bundle failed to split (Fig 9D), and two cases in which both 12A hemilineages in a segment projected their initial neurite bundles to the same side of the animal (Fig 9F).

## Discussion

### Decreased developmental robustness of hemilineage 12A in laboratory strains of *Drosophila*

The 12A neurons exhibited striking developmental variability. One common variant was the failure of the primary neurite bundle of the 12A cluster in T2 to split into dorsal and intermediate bundles. This phenotype is caused when late-born neurons, which typically take an intermediate path (Fig 5D), take a dorsal route characteristic of their earlier-born cousins (Fig 5E). However, these developing neurites still reach their appropriate target zones, although via a circuitous route. Developmental compensation of this type has been shown previously in cases where surgical manipulations forced sensory axons to reach their target area via alternative routes [42–44] and has been observed in thalamocortical connections in the mouse misrouted due to a guidance receptor mutation [45]. We show here that developmental compensation also functions in interneurons to limit the potential effects of naturally occurring pathfinding variability on connectivity. The high variability of 12A neurons provides an opportunity for investigating the underlying mechanisms of this compensation.

One notable aspect of bundle-splitting variation was that it was discontinuous, as neurons within a hemilineage covaried, resulting in all-or-none phenotypes with few exceptions. Developing neurons may be influencing each other or relying on pioneer neurons for guidance decisions. One attractive alternative possibility is that neurons within a hemilineage are inheriting a common, stochastic epigenetic state that influences their pathfinding. We have previously shown that a stochastic, repressive epigenetic state of an enhancer can be inherited by all of the neurons originating from a common neuroblast [46], lending support to this possibility.

The second common variant was ectopic branching in T2, which was due to ectopic contralateral projections from 12Ala and 12Alc neurons (Fig 5F). These variants do not appear to be corrected, so they probably result in ectopic synapses with contralateral targets, although we could not directly address connectivity with our current tools. Ectopic branching differed from bundle splitting because there was a bias toward symmetry. This left/right coordination indicates there is also a source of variation in the cellular environment that interacts with the cell-intrinsic source.

We saw variability—including fluctuating asymmetry—in both bundle splitting and ectopic branching in all of the strains we analyzed, with atypical 12A morphologies increasing in frequency as rearing temperature increased. These data strongly suggest that neurodevelopmental variability is inevitable in Drosophila, even in circuits expected to have highly genetically determined connectivity, such as those for motor patterns. This is in contrast to the alternative possibility that all neurodevelopmental variation, at least in the VNC, results from abnormal genetic mutation. Indeed, it is not unusual to find low-penetrance mutant phenotypes that are sensitive to temperature in Drosophila. However, we saw temperature-sensitive variability in four independently derived strains—two of which were recently derived from the wild in locations separated by ~8000 km. Thus, if 12A variability is not “normal” and is instead caused by specific, segregating alleles, these alleles and their phenotypic consequences must be common in nature.

Our conclusion that 12A variability is a natural feature of its underlying genetic architecture raises the question of what mechanism underlies this variability. It has recently been shown that temperature affects gene expression canalization, with 18°C being relatively canalized and higher temperatures being less canalized [47]. We propose that neuronal guidance mechanisms are sensitive to transcriptional noise, and increasing rearing temperature may be affecting 12A development by destabilizing transcriptional networks or epigenetic mechanisms.

We also found that laboratory strains were more influenced by temperature than those that were recently derived from the wild. Studies of an identified interneuron in the locust also found higher variability in laboratory strains than wild strains [48]. It may be that laboratory strains are more sensitive to temperature because they have a compromised ability to buffer against temperature-induced noise, perhaps because of relaxation of stabilizing selection pressures in the laboratory.

### Evidence for a variability “hotspot” in the *Drosophila* VNC

An intriguing finding of this study is that developmental variability is not similar across all neural elements, but, rather, there are variability “hot-spots”. Comparison of the 12A neurons in T2 with the lineage 11 neurons in the same segment showed that the latter were stable in their morphology, despite the variations in the neighboring 12A cells. Similarly, the T1 homologs of the 12A neurons show only traces of the variability that characterizes the cells in T2, despite the homology between the early guidance decisions of 12A neurites in T1 and T2. The T1 and T2 neurons of the 12A cluster are known to differ in their expression of *Ubx*, and we showed that variability could be induced in segment T1 by ectopic Ubx expression. Moreover, in the study by Marin et al. [32](E. Marin, personal communication), the loss of Ubx function in T2 reduced variability such that the neurite bundle always split (n = 13). Hence, 12A variability is both segment-dependent (due to Ubx) and lineage—dependent.

The Ubx result provides further support that the extreme variability of the 12A neurons in T2 is not simply due to low-penetrance, deleterious alleles. Mutations that directly affect axon guidance (e.g., a temperature sensitive mutation in a guidance receptor) would be expected to affect all segmental homologs, but only the T2 homologs show this variability. Thus it is either the case that the 12A neurons in T2 use different guidance receptors than the 12A neurons in T1 to make essentially identical early guidance decisions, or T1 and T2 differ in the robustness of a mechanism common to both. Regardless, our data show that Ubx is responsible for the hypervariability hot-spot in T2. Intriguingly, a high level of Ubx expression in these neurons promotes their death as seen in segment T3 [32]. We thought that levels of Ubx that were too low to evoke a cell death response might nevertheless provide some level of cellular destabilization that would be manifest in the enhanced variability shown by these cells. We tried an obvious test of this idea by blocking the apoptotic pathway by expressing two different inhibitors of apoptosis, p35 and Diap1, in the T2 neurons to see if this reduced the developmental variability in the T2 cells, but their levels were typical of laboratory strains (unpublished). This suggests Ubx promotes variability at low levels independent of its role in promoting cell-death at high levels. It may be that the low levels of Ubx that specify T2 fate promote transcriptional instability in a manner analogous to temperature.

One important implication of our finding that Ubx promotes hypervariability, and that strains quantitatively vary in the level of this hypervariability, is that neurodevelopmental variability can be treated as a genetically controlled, quantitative trait in Drosophila. Thus we can use Drosophila, and the easy-to-score 12A phenotype in particular, to identify and characterize the genetic mechanisms that promote neurodevelopmental robustness. We find this to be an exciting possibility, as characterization of these mechanisms will be instructive for thinking about genetic risk for neurodevelopmental disorders in human populations [8] and could possibly identify gene pathways for candidate therapeutic targets. In the near term, it will be important to ask whether strain variation in Ubx-dependent hypervariability is mediated by variation in key developmental genes, by alleles of small effect distributed throughout the genome, or by mutations in genes known to promote phenotypic robustness in other contexts (e.g. HSP90 [6]).

Why might the 12A hemilineage in T2 be a particularly variable population of neurons, even in wild-derived flies? We suspect that it may have something to do with the involvement of 12A neurons in the production of courtship wing song. It is well appreciated that courtship song is highly diverse among the *Drosophila* species [49]. It is possible that at least some of this diversity may be due to changes in neuronal connectivity within the networks that produce song. Perhaps the developmental programs responsible for 12A development must be somewhat developmentally unstable to remain evolutionarily flexible. Alternatively, loss of developmental canalization may accompany rapid, adaptive evolution of wing song or flight [50].

### Additional examples of neurodevelopmental variability

Our findings of developmental variability were not limited to 12A in T2. We also identified a fairly common variant in the production of a novel intermediate commissure in T1, which was mostly limited to animals that were heterozygous or homozygous for the HI genotype. This variant was unique in that the frequency of ectopic T1 branching in the HI genotype was consistent across temperatures and was always symmetrical. Notably, the phenotype frequencies in HI, CT/HI, and HI/CT indicate this phenotype is dominant and thus amenable to genetic analysis. Preliminary crossing experiments suggest that the inheritance of the variant is complex and may be polygenic or monogenic with incomplete penetrance (unpublished data). This developmental variant is particularly notable given the role of the 12A neurons in T1 in wing song, which is a rapidly evolving trait among *Drosophila* species.

The other developmental variants we observed included duplicated neuroblasts and major pathfinding variants. These events were distributed across multiple segments, not restricted to the hypervariable T2 hemilineage. Thus it is probably fair to extrapolate our findings here to the whole CNS, which implies that developmental noise affects Drosophila nervous system development at a high frequency. For example, 7 out of 520 lineage 12 neuroblasts were duplicated in Canton S larvae raised at 16°C. If a 1.3% frequency holds over the approximately 500 neuroblasts found in larvae, then essentially every Canton S larva raised at 16°C would be expected to have at least one duplicated neuroblast somewhere in its nervous system.

We point out, however, that not all variability increased with temperature: neuroblast duplications were most frequent at low temperatures in the T/CS genotype. Thus, the effect of temperature on nervous system development is complex and may be specific to different developmental mechanisms.

Considering developmental noise is probably common in the Drosophila CNS, it makes sense that the nervous system can accommodate variation such that core functions remain intact. One example of a mechanism to limit the effects of developmental variation comes from the visual system, where mutual inhibition ensures robust patterns of connectivity despite variable numbers of neurons [18]. The same mechanism may function throughout the nervous system to accommodate variable neuron numbers, and may allow the nervous system to readily accommodate the extra neurons produced by neuroblast duplications. This hypothesis should be testable, perhaps by working in the T/CS genetic background, or by genetically inducing neuroblast duplications and following their development.

### Potential behavioral effects of neurodevelopmental variability

Behavioral differences between individuals may be in part due to differences in neural network connectivity that arise during development. In this study, flies exhibited interindividual variation when spontaneously initiating flight, and this variation was bimodal, with the frequency of flies initiating flight in the early mode correlating with the frequency of typical 12A development across strains. Given 12A neurons are involved in wing control, we hypothesize that aberrant 12A development causes the delay, perhaps by impeding the ability of descending interneurons from initiating a flight central pattern generating circuit. Alternatively, delayed flight initiation and ectopic branching could both be dependent on a common latent variable, *e*.*g*. a general increase in both developmental and behavioral variability in laboratory strains of flies as compared to those found in nature. However, we didn’t observe a correlation with the unsplit phenotype, despite the unsplit phenotype also being less frequent in the wild strains.

The correlation between delayed flight initiation and 12A variability, when combined with our developmental data, lead us to hypothesize that decades of laboratory rearing have selected for a reluctance to initiate flight, and that this selection pressure has acted on a variability “hot-spot” in the VNC flight circuitry, leading to a destabilized developmental program in the 12A neurons of T2.

## Supporting Information

S1 Fig Simulated effect of differentially mixed populations on distribution of behavior data. R simulation of how two populations (n = 40) that differ in the frequency of animals with the ectopic branch phenotype might look if the ectopic branch phenotype produces a 3σ effect. In conditions in which only 8% of animals harbor the ectopic branch phenotype (*e*.*g*. HI) the data are distributed normally with only a few outliers. In conditions in which 50% of the animals harbor the phenotype (*e*.*g*. T/OR), a pronounced second mode is expected, resulting in a much larger spread to the data as compared to the 8% condition.
(PDF)

S2 Fig Repeat of spontaneous flight initiation experiment confirms correlation between late takeoff and ectopic branch frequencies. Numbers of animals for each proportion of animals initiating flight in late mode—*CT*, *16*: 13; *CT*, *29*: 11; *HI*, *16*: 16; *T/CS*, *16*: 20; *T/CS*, *29*: 8; *T/OR*, *16*: 25; *T/OR*, *29*: 21.
(PDF)

S3 Fig No overt effect of 12A variability on wing song or escape from a looming stimulus. (**A-G**) Effects of genotype and rearing temperature on various parameters of wing song. In no case do both the T/CS and T/OR groups show a greater spread to the data than in both the CI and HI groups. This indicates that any potential effect of ectopic branching is small. (**H**) Effect of genotype and rearing temperature on the time it takes for a fly to initiate an escape response following presentation of a looming stimulus. As with wing song, there is no clear effect of ectopic branching.
(PDF)

S1 Table Raw Data—T2 branch variation. Full dataset of scores of bundle splitting and ectopic branch phenotypes for all animals in this study. A “1” indicates the presence of the phenotype. Tab-delimited text.
(TXT)

S2 Table Raw Data—Freefall recovery. Data used in the creation of Fig 8E. Tab-delimited text.
(TXT)

S3 Table Raw Data—Voluntary takeoff delay. Data used in the creation of Fig 8A. Tab-delimited text.
(TXT)

S4 Table Raw Data—Elicited takeoff delay. Data used in the creation of S3 Fig panel H. Tab-delimited text.
(TXT)

S5 Table Raw Data—Courtship Song. Data used in the creation of S3 Fig panels A-G. Tab-delimited text.
(TXT)
