# Supplementary figures and images for: Genetic and Environmental Control of Neurodevelopmental Robustness in Drosophila
Source: PLoS One. 2016 May 25;11(5):e0155957. doi: 10.1371/journal.pone.0155957 (PMC4880190; doi:10.1371/journal.pone.0155957)

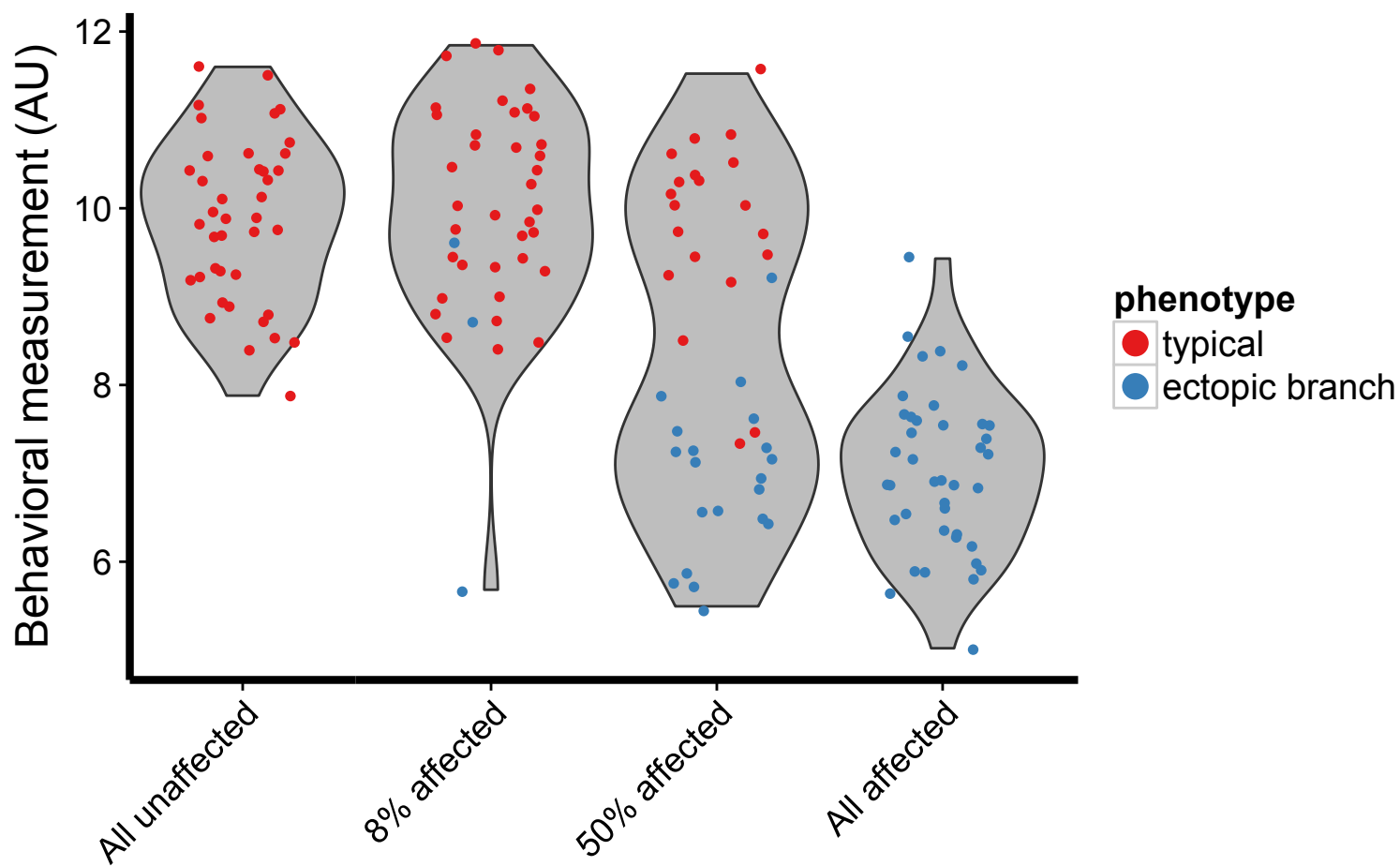

Supplement: S1 Fig — R simulation of how two populations (n = 40) that differ in the frequency of animals with the ectopic branch phenotype might look if the ectopic branch phenotype produces a 3σ effect. In conditions in which only 8% of animals harbor the ectopic branch phenotype (e.g. HI) the data are distributed normally with only a few outliers. In conditions in which 50% of the animals harbor the phenotype (e.g. T/OR), a pronounced second mode is expected, resulting in a much larger spread to the data as compared to the 8% condition. (PDF) [file pone.0155957.s001.pdf]

## Pearson correlation

$r = 0.81$   
 $P = 0.027$

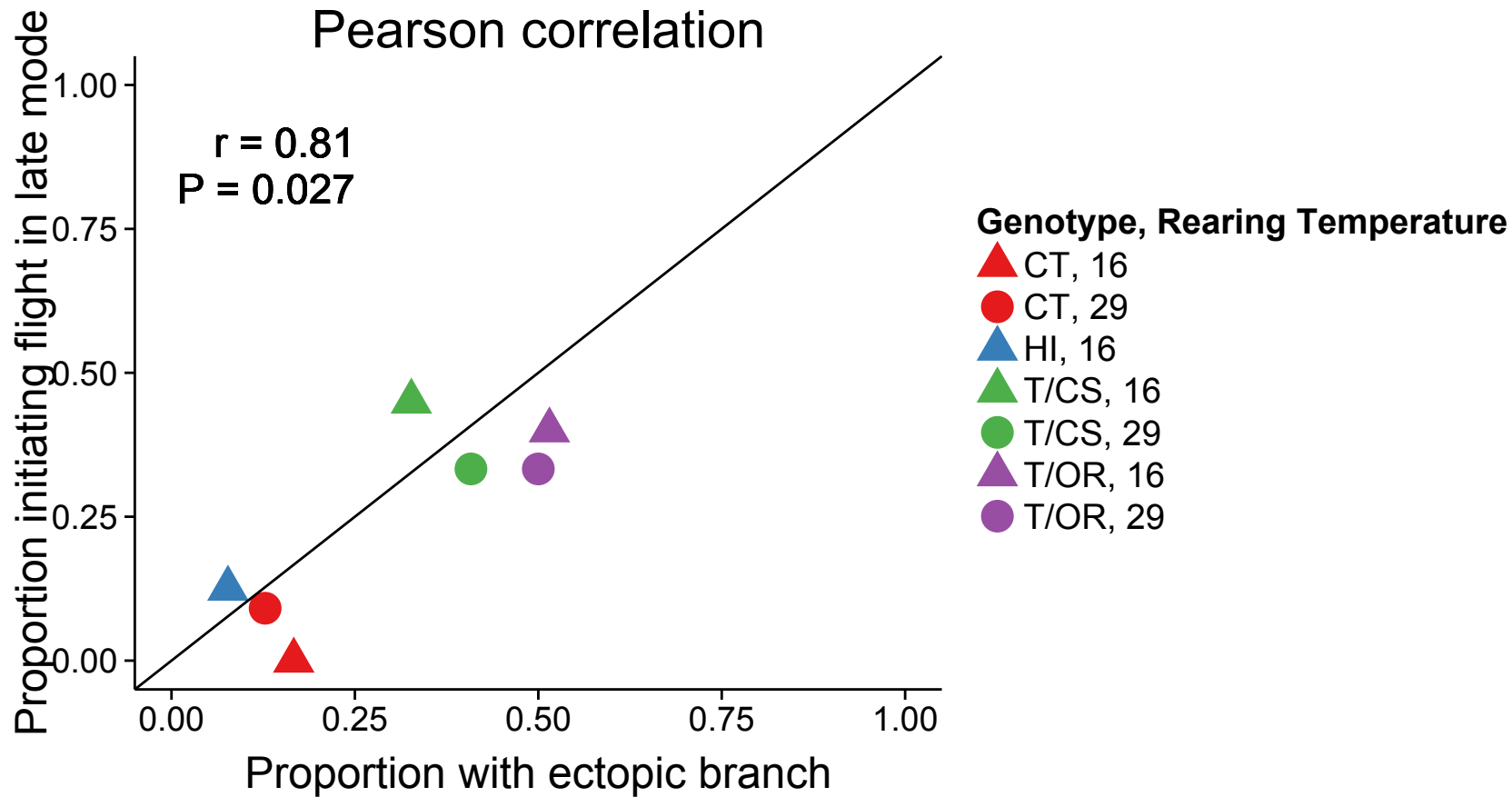

Supplement: S2 Fig — Numbers of animals for each proportion of animals initiating flight in late mode—CT, 16: 13; CT, 29: 11; HI, 16: 16; T/CS, 16: 20; T/CS, 29: 8; T/OR, 16: 25; T/OR, 29: 21. (PDF) [file pone.0155957.s002.pdf]

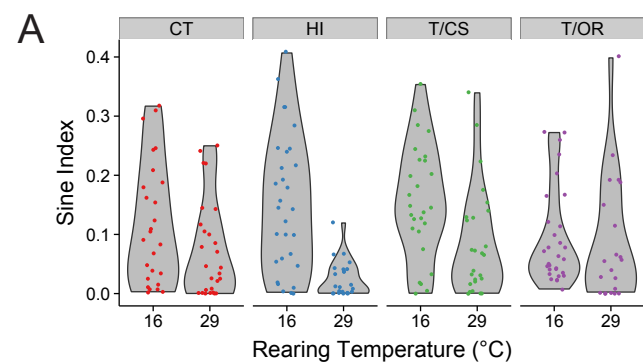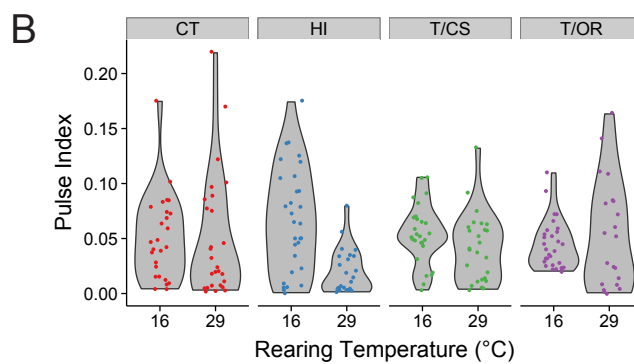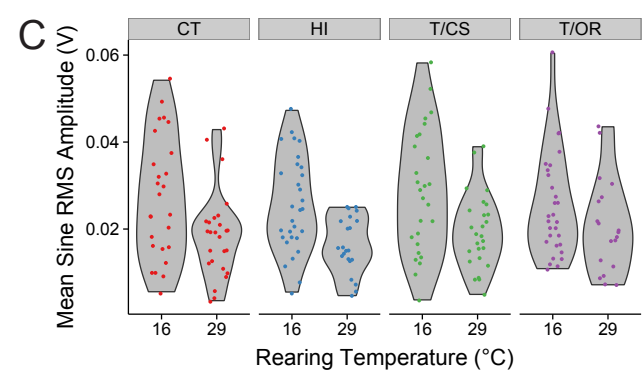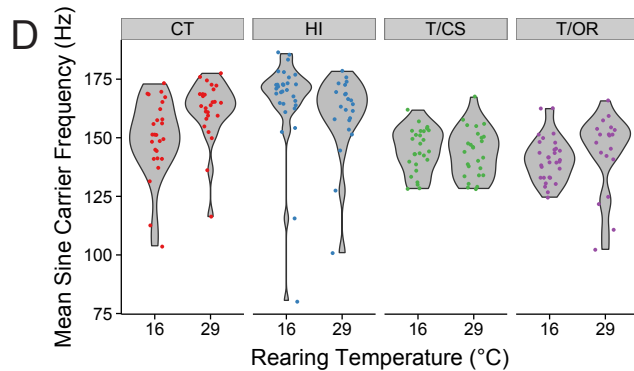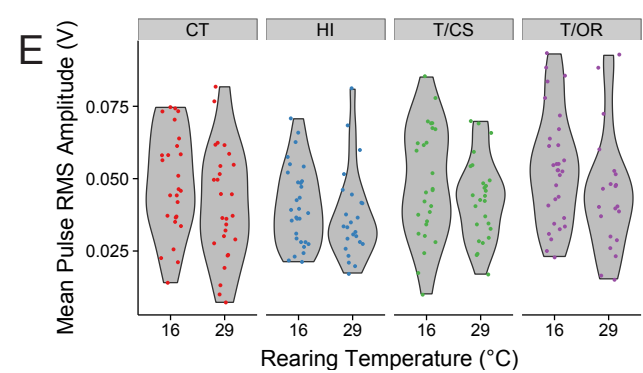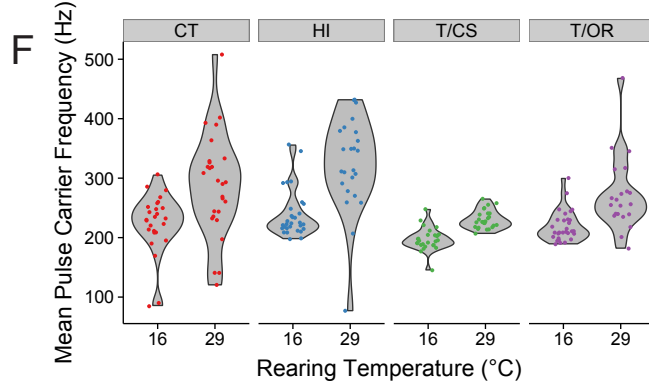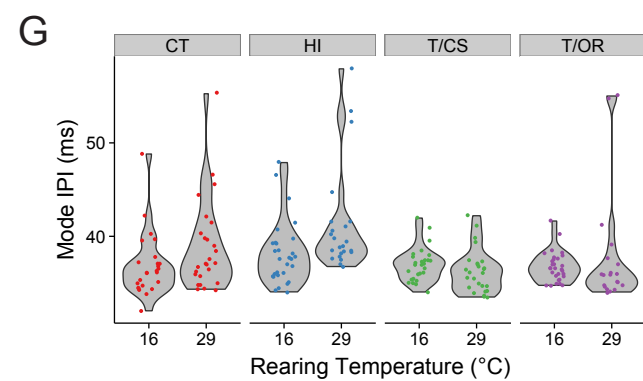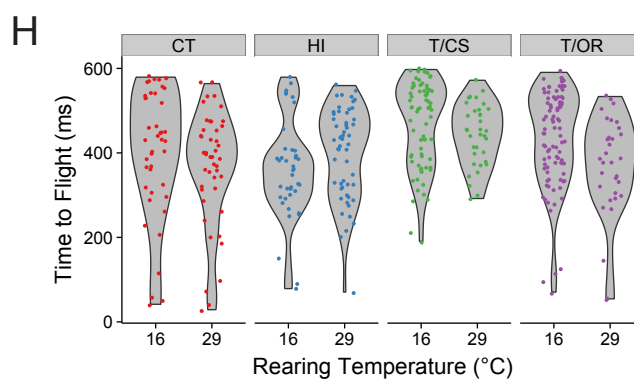

Supplement: S3 Fig — (A-G) Effects of genotype and rearing temperature on various parameters of wing song. In no case do both the T/CS and T/OR groups show a greater spread to the data than in both the CI and HI groups. This indicates that any potential effect of ectopic branching is small. (H) Effect of genotype and rearing temperature on the time it takes for a fly to initiate an escape response following presentation of a looming stimulus. As with wing song, there is no clear effect of ectopic branching. (PDF) [file pone.0155957.s003.pdf]
